# Killing with proficiency: Integrated post-translational regulation of an offensive Type VI secretion system

**DOI:** 10.1371/journal.ppat.1007230

**Published:** 2018-07-27

**Authors:** Adam Ostrowski, Francesca R. Cianfanelli, Michael Porter, Giuseppina Mariano, Julien Peltier, Jun Jie Wong, Jason R. Swedlow, Matthias Trost, Sarah J. Coulthurst

**Affiliations:** 1 Division of Molecular Microbiology, School of Life Sciences, University of Dundee, Dundee, United Kingdom; 2 Centre for Gene Regulation and Expression, School of Life Sciences, University of Dundee, Dundee, United Kingdom; 3 Institute for Cell and Molecular Biosciences, Newcastle University, Newcastle-upon-Tyne, United Kingdom; 4 MRC Protein Phosphorylation and Ubiquitylation Unit, School of Life Sciences, University of Dundee, Dundee, United Kingdom; Swiss Federal Institute of Technology Lausanne (EPFL), SWITZERLAND

## Abstract

The Type VI secretion system (T6SS) is widely used by bacterial pathogens as an effective weapon against bacterial competitors and is also deployed against host eukaryotic cells in some cases. It is a contractile nanomachine which delivers toxic effector proteins directly into target cells by dynamic cycles of assembly and firing. Bacterial cells adopt distinct post-translational regulatory strategies for deployment of the T6SS. ‘Defensive’ T6SSs assemble and fire in response to incoming attacks from aggressive neighbouring cells, and can utilise the Threonine Protein Phosphorylation (TPP) regulatory pathway to achieve this control. However, many T6SSs are ‘offensive’, firing at all-comers without the need for incoming attack or other cell contact-dependent signal. Post-translational control of the offensive mode has been less well defined but can utilise components of the same TPP pathway. Here, we used the anti-bacterial T6SS of *Serratia marcescens* to elucidate post-translational regulation of offensive T6SS deployment, using single-cell microscopy and genetic analyses. We show that the integration of the TPP pathway with the negative regulator TagF to control core T6SS machine assembly is conserved between offensive and defensive T6SSs. Signal-dependent PpkA-mediated phosphorylation of Fha is required to overcome inhibition of membrane complex assembly by TagF, whilst PppA-mediated dephosphorylation promotes spatial reorientation and efficient killing. In contrast, the upstream input of the TPP pathway defines regulatory strategy, with a new periplasmic regulator, RtkS, shown to interact with the PpkA kinase in *S*. *marcescens*. We propose a model whereby the opposing actions of the TPP pathway and TagF impose a delay on T6SS re-assembly after firing, providing an opportunity for spatial re-orientation of the T6SS in order to maximise the efficiency of competitor cell targeting. Our findings provide a better understanding of how bacterial cells deploy competitive weapons effectively, with implications for the structure and dynamics of varied polymicrobial communities.

## Author summary

Pathogenic bacteria use a weapon called the ‘Type VI secretion system’ (T6SS) to fire toxic proteins into other cells. These target cells are very often rival bacteria, allowing the T6SS-wielding bacteria to outcompete other pathogens or harmless bacteria. Some bacteria deploy this weapon defensively, only firing back at aggressive cells that have attacked them. Many others use it offensively, firing their T6SS pre-emptively, irrespective of whether they have been attacked by other cells. This work aimed to discover how the activity of the T6SS is controlled in offensive bacteria compared with defensive bacteria, and how this regulation facilitates efficient killing of targeted bacteria. To do this, we studied the offensive anti-bacterial T6SS of *Serratia marcescens*, a pathogen causing hospital-acquired infections. We found that offensive and defensive bacteria use similar regulatory pathways to activate their T6SSs efficiently, by controlling assembly of the basic machinery and allowing time for the machine to re-position around the cell between firing events. However, the sensing proteins feeding into the regulatory pathways are different and we were able to identify a new offensive input protein. Our findings provide a better understanding of how bacteria can kill competitors, whether aggressive or peaceful neighbours, so effectively.

## Introduction

Bacteria constantly face a challenging external environment, with their survival dependent on the ability to adapt to abiotic conditions, overcome host defences or successfully compete against rival bacterial cells. Critical to all of these is the use of sophisticated protein secretion systems to translocate specific proteins across the bacterial envelope to the cell exterior, extracellular milieu or directly into other cells [1]. The Type VI secretion system (T6SS) is widespread in Gram-negative bacteria and is able to deliver toxic proteins, known as effectors, directly into target cells. Whilst some T6SSs are deployed by pathogenic bacteria against host eukaryotic organisms as direct virulence factors, it is now believed that the majority of T6SSs are involved in inter-bacterial competition and represent a crucial factor in conferring a fitness advantage in a variety of polymicrobial niches [2–4]. Many important pathogens, in addition to commensals and environmental bacteria, use anti-bacterial T6SSs to deliver anti-bacterial toxins into rival bacterial cells, whilst protecting themselves and their siblings from intoxication through possession of cognate immunity proteins able to neutralise each T6SS-delivered effector. T6SS-dependent anti-bacterial effectors include families of peptidoglycan hydrolases, phospholipases and DNases, in addition to examples of pore forming toxins, NAD-glycohydrolases and others of currently unknown function [2, 4–6]. The T6SS can promote very efficient killing of competitors and its importance in maintaining and disrupting complex communities such as the human gut microbiota is becoming increasingly appreciated [7, 8].

The T6SS is a trans-envelope nanomachine related to a contractile bacteriophage tail. According to the current model for the T6SS core machinery [9–14], the T6SS propels a puncturing structure, comprising a tube of stacked Hcp hexamers tipped with a VgrG-PAAR spike, out of the secreting cell towards a target cell or into the medium. This expelled puncturing structure is decorated with effector proteins by covalent and non-covalent interactions, mediating effector translocation into the target cell. The T6SS is assembled from a membrane complex (TssJLM) which then docks a cytoplasmic baseplate structure (TssEFGK, VgrG-PAAR), to form the basal complex. The Hcp tube and a surrounding contractile sheath (TssBC) then assemble from the baseplate and extend into the cytoplasm. Subsequently, rapid and powerful contraction of the TssBC sheath ‘fires’ the Hcp-VgrG-PAAR structure out of the secreting cell, followed by TssH-mediated disassembly of the contracted sheath. Fluorescence microscopy has allowed visualisation of dynamic cycles of T6SS assembly, contraction and recycling in several organisms [12, 15–17]. Additionally, in *P*. *aeruginosa*, it revealed that T6SS assembly is spatially regulated to occur in response to an incoming attack by the T6SS of a neighbouring cell [18]. This regulation is mediated by T6SS accessory proteins forming a post-translational regulatory system called the Threonine Protein Phosphorylation (TPP) pathway [18–22]. An incoming attack is sensed by the TagQRST complex, activating a membrane-bound threonine kinase, PpkA, to phosphorylate Fha1. Fha1 phosphorylation promotes assembly of an active T6SS at the site of incursion and is antagonised by the phosphatase, PppA. The outcome of this regulation is that the *P*. *aeruginosa* T6SS exhibits ‘defensive’ behaviour, with T6SS activity only observed under conditions of close cell-cell contact with an aggressive T6SS-elaborating neighbour and not seen in liquid media or against non-aggressive competitors [18].

A defensive strategy is not, however, the only or perhaps even the most common mode of anti-bacterial T6SS deployment. Many T6SSs rather exhibit ‘offensive’ behaviour, firing without the need for cell-cell contact or incoming attack and able to attack non-attacking competitors. These include T6SSs in *Vibrio cholerae*, *Burkholderia thailandensis*, *Acinetobacter* sp. and *Serratia marcescens* [17, 18, 23]. Additionally, while many T6SS gene clusters encode the basic components of the TPP pathway (PpkA-, PppA- and Fha-like proteins), the TagQRST sensing complex is restricted to *Pseudomonas*, suggesting that the TPP pathway may be utilised differently in other organisms [19, 22, 24]. Therefore important outstanding questions concern how the TPP pathway is used more broadly and how offensive behaviour can be regulated. A further conserved accessory protein, TagF, has been implicated as a negative post-translational regulator in the defensive system [21], however the details of its integration with the TPP pathway, role in offensive systems and how it interacts with the core T6SS machinery remain to be defined. An ideal model system to address these questions is the single T6SS of *S*. *marcescens* Db10, which delivers multiple anti-bacterial effectors and has potent activity against closely- and distantly-related competitors [25, 26]. This T6SS is regulated by the TPP pathway, requiring PpkA-mediated phosphorylation of Fha for its activity, and has a TagF protein. However it is clearly an ‘offensive’ T6SS, able to fire efficiently in the absence of cell-cell contact and to effectively kill non-aggressive as well as aggressive competitors [17, 26, 27]. *S*. *marcescens* itself is a typical opportunist pathogen, found in diverse environmental communities but also able to cause problematic and antibiotic-resistant human infections, both contexts in which its ability to compete with other organisms and the host microflora will be critical [28, 29].

In this work, we describe how the TPP pathway and TagF can be integrated with the core T6SS machinery to achieve efficient post-translational control of T6SS-mediated anti-bacterial activity. We provide evidence that opposing actions of the TPP pathway and TagF on assembly of the core machinery facilitates spatial reorientation, in a mechanism shared between offensive and defensive T6SSs. In contrast, the upstream input to the TPP pathway defines regulatory strategy, with a new regulator, RtkS, directing offensive behaviour. Our findings provide an increased understanding of how bacterial cells deploy their competitive weapons to maximum effect, which in turn will define the structure and dynamics of varied polymicrobial communities.

## Results

### Phosphorylation of Fha by PpkA promotes formation of Fha foci and assembly of an active T6SS in *Serratia marcescens*

**Fig 1:**
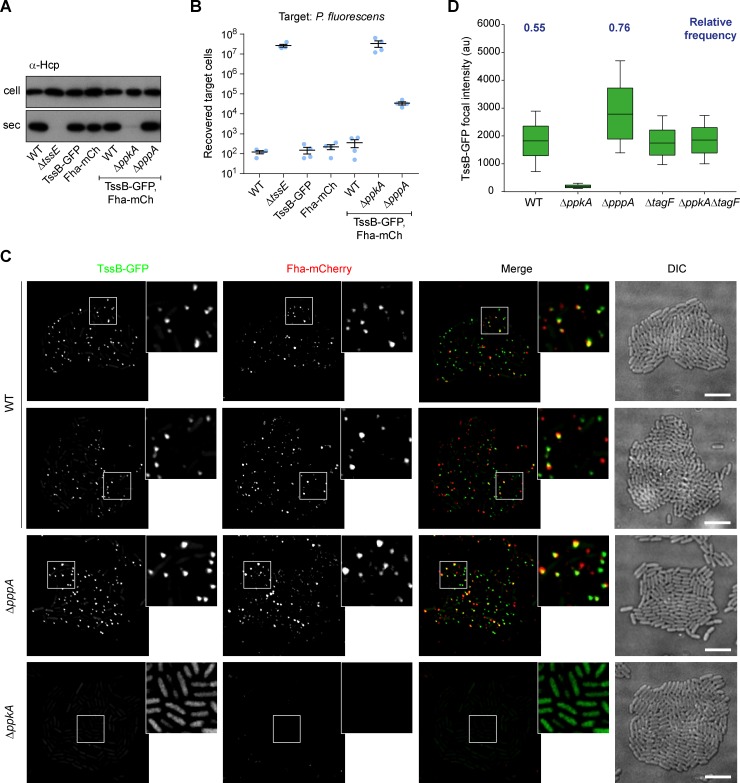
Phosphorylation of Fha promotes formation of Fha and TssB foci in *S*. *marcescens* Db10. (A) T6SS-dependent secretion of Hcp by wild type (WT) *S*. *marcescens* Db10 or mutants lacking the kinase PpkA (Δ*ppkA*) or the phosphatase PppA (Δ*pppA*), and by derivatives expressing fusions of GFP to the C-terminus of TssB (TssB-GFP) and mCherry to the C-terminus of Fha (Fha-mCherry), in an otherwise wild type, Δ*ppkA* or Δ*pppA* mutant background. The T6SS inactive mutant Δ*tssE* is a negative control and cellular and secreted fractions were subjected to immunoblotting using anti-Hcp antisera. (B) T6SS-dependent anti-bacterial activity of fluorescent reporter strains against a *P*. *fluorescens* target. Number of recovered target cells following 4 h co-culture with wild type or mutant strains of *S*. *marcescens* Db10 as indicated. Individual data points are overlaid with mean +/- SEM (n = 4). (C) Representative images of cells expressing TssB-GFP and Fha-mCherry fusions, in a wild type, Δ*ppkA* or Δ*pppA* mutant background. From left to right, panels show individual fluorescence channels (TssB-GFP, Fha-mCherry), an overlay of the fluorescent channels (Merge; GFP signal false-coloured green and mCherry red), and the corresponding DIC image. Inset panels show a zoomed-in view of the region represented by the white box; for the Δ*ppkA* mutant, the contrast has been increased in the inset panels to reveal non-focal TssB-GFP fluorescence. Scale bars, 5 μm. (D) Fluorescent signal intensity measurement of the segmented TssB-GFP signal in wild type, Δ*ppkA*, Δ*pppA*, Δ*tagF* and Δ*ppkA*Δ*tagF* mutants (au, arbitrary fluorescence units). The boundaries of the boxes represent the 25 and 75 percentiles and the horizontal lines are the median values of the analysed population. The whisker bars represent standard error (10 and 90 percentiles). Regions of interest from at least 32 fields of view were analysed in each case. Relative frequency of TssB-GFP foci (expressed per cell at a single timepoint) were estimated by combining automated focus detection with manual cell counting in a representative 15 of these images.

We have previously reported that phosphorylation of Fha by PpkA is required for Hcp secretion, effector secretion and T6SS-mediated anti-bacterial activity in *S*. *marcescens* Db10, and that phosphorylation is reversed by PppA [27]. To observe the impact of this post-translational regulatory pathway at the single cell level, a fusion of Fha with C-terminal mCherry (Fha-mCherry) was introduced into *S*. *marcescens* Db10 at the normal chromosomal location. This fusion was fully functional, since its introduction had no impact on Hcp secretion or anti-bacterial activity compared with the wild type strain (Fig 1A and 1B). An assembled and firing T6SS can be observed through the detection of bright TssB-GFP foci corresponding to polymerised TssBC sheaths in the extended (pre-firing) and contracted (post-firing) forms [15, 17]. Therefore to be able to link Fha focus formation with T6SS firing events, we combined the Fha-mCherry fusion with a TssB-GFP fusion and subsequently further introduced in-frame kinase (Δ*ppkA*) or phosphatase (Δ*pppA*) deletions. Introduction of the fluorescent fusion proteins had no impact on Hcp secretion or anti-bacterial activity compared with the equivalent non-fluorescent strain, and the integrity and similar total abundance of the fusion proteins in each background was confirmed by immunoblotting (Fig 1A and 1B, S1 Fig).

Observing single cells by fluorescence microscopy revealed not only the formation of bright TssB-GFP foci in the wild type background, as previously [17], but also the formation of distinct foci of Fha-mCherry (Fig 1C, S2 Fig). In many cases the Fha-mCherry foci appeared to be co-localised with the TssB-GFP foci, but examples of Fha-mCherry foci without corresponding TssB-GFP foci, and of TssB-GFP without visible Fha-mCherry, were also readily apparent. Formation of both TssB-GFP and Fha-mCherry foci was completely eliminated in a Δ*ppkA* mutant (Fig 1C) and could be restored by expression of PpkA *in trans* (S2 Fig). This implies that phosphorylation of Fha is required for both Fha focus formation and assembly of a firing-competent T6SS and is consistent with the lack of Hcp secretion in Δ*ppkA* (Fig 1). In contrast, foci of both TssB and Fha were present, at least as numerous, and apparently brighter in the Δ*pppA* mutant compared with the wild type (Fig 1C). We have reported previously [27] and show again here that levels of Hcp secretion in the Δ*pppA* background are at least as high as in the wild type, whereas efficiency of anti-bacterial activity is significantly reduced (Fig 1).

In order to determine the impact of Δ*pppA* and other mutations on the formation of Fha and TssB foci in a quantitative manner, we aimed to implement automated focus detection and analysis of a large number of cells. The GFP signal within each region of interest was segmented to generate a binary mask, the mean fluorescence intensity within each identified focus was measured, and the distribution of mean focal intensities was determined in each genetic background. Simultaneously, automated TssB focus detection was combined with manual cell counting in 15 randomly-selected images of the wild type and the Δ*pppA* mutant (total of 1597 and 1913 cells, respectively) to estimate frequency of focus formation. In the wild type background, the frequency of TssB-GFP foci, expressed per cell at a single instant, was 0.55, whereas in the Δ*pppA* mutant the frequency of focus formation increased to 0.76. In agreement with visual inspection, we observed a significant increase in the TssB-GFP signal intensity in the Δ*pppA* strain (median = 2780) compared with the wild type (median = 1822) (ANOVA on ranks P < 0.05; Fig 1D). In the absence of real foci in the Δ*ppkA* mutant, the detection algorithm identified regions of diffuse cellular fluorescence in some of the cells, but the fluorescence intensity values were much lower than those obtained from images displaying foci. Unfortunately, due to the fluorescent signal intensity and signal-to-noise ratio being considerably lower for the Fha-mCherry foci than TssB-GFP, it was not possible to successfully perform automated focus detection and downstream quantitative analyses involving this protein. However our visual observation, through examination of multiple images, that the intensity of Fha-mCherry foci appears to increase in a Δ*pppA* mutant compared with the wild type is in agreement with the increased intensity of Fha-mCherry foci reported previously in the Δ*pppA* mutant of *P*. *aeruginosa* [20].

### PppA-mediated dephosphorylation promotes spatial re-orientation of the T6SS

**Fig 2:**
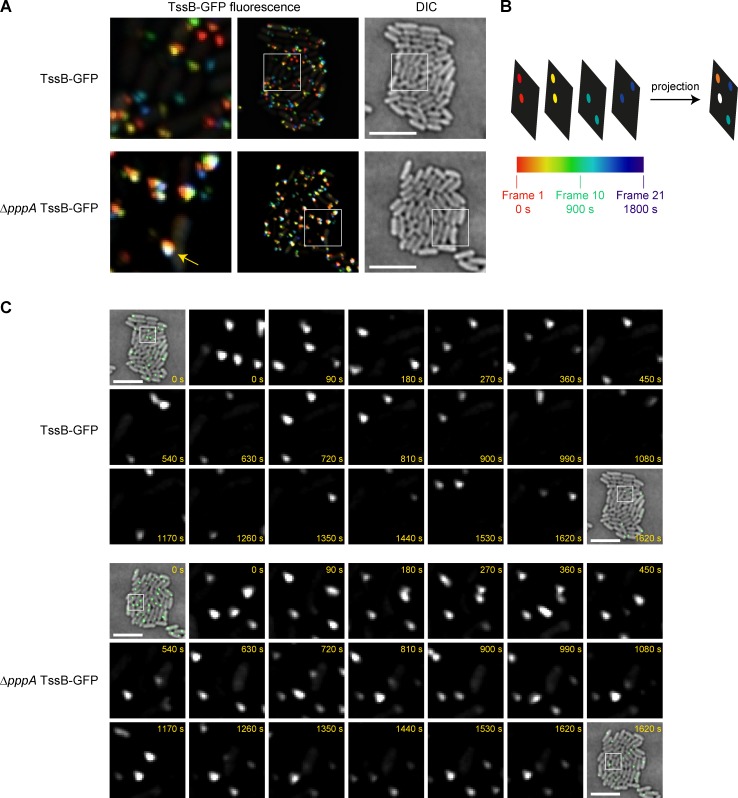
Loss of PppA limits spatial redistribution of the T6SS apparatus. (A) Localisation of TssB-GFP in the wild type or Δ*pppA* mutant of *S*. *marcescens* Db10 was observed over a 30 min period, with image acquisition every 90 s, and a representative time-lapse sequence of each is shown. The fluorescent signal in individual frames is colour coded by time according to the scale shown in part B and the frames are superimposed. Left and centre image panels, fluorescence projections with the region indicated by the white box in the centre panel being magnified in the left panel; right image panel, corresponding DIC image. The yellow arrow indicates an example of a white coloured focus of TssB-GFP readily visible in composite images from the Δ*pppA* strain. A further seven fields of view for each strain are presented in Supporting Information S3 Fig. (B) Schematic illustration of how fluorescence projection images were generated. (C) Magnified sections from individual frames of the same time-lapse sequences as part A. First and last panels show GFP signal false-coloured in green and overlaid on the corresponding DIC image of the microcolony, other panels show the TssB-GFP fluorescence signal from the area indicated by the white boxes. The acquisition time in seconds is indicated. (A, C) Scale bar, 5 μm. The full time-lapse sequences including DIC are presented in Supporting Information S4 Fig.

The observation that a Δ*pppA* mutant has no impairment in secretion to the medium but is less efficient than the wild type in targeting other bacterial cells is consistent with the idea that PppA-mediated dephosphorylation of Fha is required for partial disassembly and re-assembly of the T6SS at a new location in the cell, as proposed previously [17, 18]. To investigate this hypothesis at the single cell level, TssB-GFP foci dynamics were studied over a period of 30 minutes. This revealed a high turnover of the TssB-GFP foci from one position to another in the wild type strain, while many of the foci in the Δ*pppA* strain appeared to be more static (Compare S1 Movie with S2 Movie). To provide a more quantitative representation of this phenomenon, temporal projections of eight representative time-courses for each strain were generated, with different colours assigned to the fluorescent signal observed at each timepoint (Fig 2A and 2B, S3 Fig). An RGB scale was used such that the first frame is represented by red colour and the last frame by blue. Blending of pixel colour towards white as the frames are superimposed indicates that the given pixel was fluorescent for multiple time points throughout the experiment. In this analysis, the majority of the TssB-GFP signal in the wild type strain was represented by various colours, indicating that the T6SS is rapidly turned over and reassembled at different sites within the cell. In contrast, the Δ*pppA* mutant displayed a significant number of white spots, indicating that TssB-GFP foci were detected in these locations over many time points (Fig 2A, S3 Fig). The TssB-GFP foci appearing as white spots in projected images of the Δ*pppA* mutant were further examined frame-by-frame. This revealed that almost all of these foci, whilst present at the same position for long periods during the time course, could be seen to markedly diminish and/or disappear entirely for at least one frame (Fig 2C, S4 Fig). This evidence suggests that in the absence of PppA, spent and disassembled TssBC sheath tends to be promptly re-assembled at the same site in the cell, whereas in wild type cells the T6SS assembly site changes rapidly.

### TagF is a negative regulator of T6SS assembly

**Fig 3:**
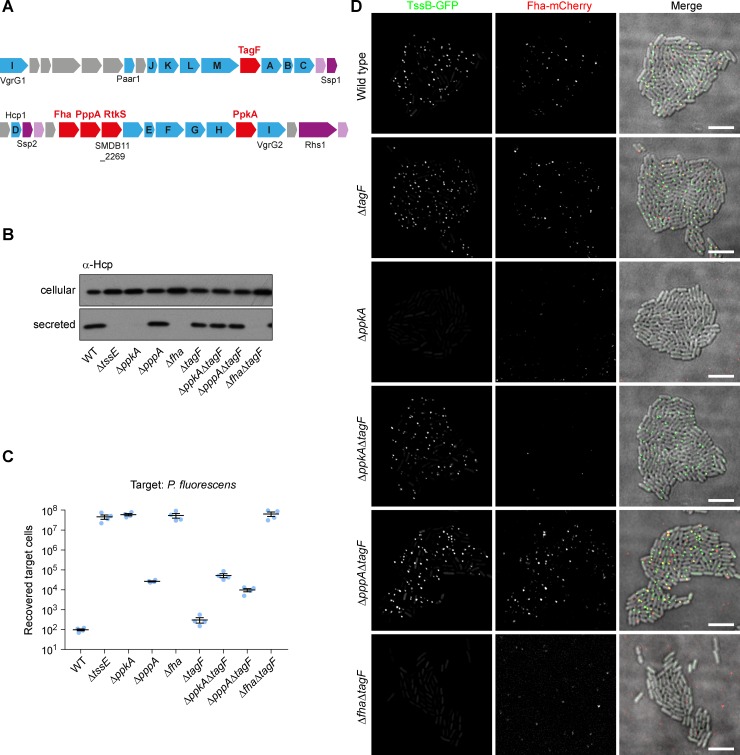
Phosphorylation of Fha and formation of Fha foci is no longer required for T6SS activity in the absence of the post-translational regulator TagF. (A) Schematic depiction of the T6SS gene cluster of *S*. *marcescens* Db10 showing the genes encoding components of the T6SS post-translational regulatory system in this organism (in red). Core T6SS components are shown in blue, with letters indicating TssA-M, and effector/immunity pairs are shown in purple/pink. (B) T6SS-dependent Hcp secretion as measured by immunoblot detection of Hcp in cellular and secreted fractions of wild type (WT) or mutant (Δ*tssE*, Δ*ppkA*, Δ*pppA*, Δ*fha*, Δ*tagF*, Δ*ppkA*Δ*tagF*, Δ*pppA*Δ*tagF* and Δ*fha*Δ*tagF*) strains of *S*. *marcescens* Db10. (C) T6SS-dependent anti-bacterial activity as determined by recovery of target organism *P*. *fluorescens* following co-culture with wild type or mutant strains of *S*. *marcescens* Db10. Individual data points are overlaid with mean +/- SEM (n = 4). (D) Representative images of wild type and mutant strains of *S*. *marcescens* Db10 expressing TssB-GFP and Fha-mCherry fluorescent fusions. Panels show individual fluorescence channels (TssB-GFP, Fha-mCherry) and an overlay of the fluorescence channels and the DIC channel (Merge; GFP signal false-coloured green and mCherry red). Additionally, TssB-GFP was monitored in the Δ*fha*Δ*tagF* background. The mCherry channel image in this case illustrates the low level but frequently observed background signal unrelated to mCherry expression. Scale bar, 5 μm.

It has been reported previously that TagF is a post-translational negative regulator of the defensive T6SS in *P*. *aeruginosa* [21]. However the impact of TagF on sheath assembly and Fha focus formation at the single cell level, and on the overall activity of the offensive T6SS of *S*. *marcescens*, has not yet been reported. A TagF homologue, SMDB11_2256, is encoded immediately downstream of TssM in the T6SS gene cluster of *S*. *marcescens* Db10 (Fig 3A). Mutants carrying in-frame deletions of *tagF* in wild type, Δ*ppkA*, Δ*pppA* and Δ*fha* backgrounds were constructed and their T6SS activity assessed. The Δ*tagF* mutant showed no significant difference in Hcp secretion and T6SS-mediated anti-bacterial activity compared with the wild type (Fig 3B and 3C), consistent with loss of a negative regulator in a system that is already ‘on’. However, introduction of Δ*tagF* into a Δ*ppkA* background had a large impact, restoring full Hcp secretion and considerable anti-bacterial activity to the otherwise-inactive Δ*ppkA* kinase mutant (compare Δ*ppkA*Δ*tagF* and Δ*ppkA*, Fig 3B and 3C). Introduction of Δ*tagF* into the Δ*pppA* phosphatase mutant did not affect Hcp secretion and resulted in a minor increase in anti-bacterial activity. Since loss of TagF was able to overcome the requirement for PpkA, and thus phosphorylation of Fha, in T6SS activity, the requirement for Fha itself in the absence of TagF was determined. In contrast to PpkA, loss of TagF was not able to compensate for loss of Fha, since the T6SS of the Δ*fha*Δ*tagF* mutant was inactive (Fig 3B and 3C). Thus Fha is essential even in the absence of TagF.

Next, TagF single and double mutants were combined with the TssB-GFP and Fha-mCherry reporter fusions to examine the impact of TagF on T6SS assembly at the single cell level. In agreement with T6SS activity assays, the single Δ*tagF* mutant exhibited TssB-GFP and Fha-mCherry foci indistinguishable from the wild type (Fig 3D, Fig 1D). Similarly, the introduction of Δ*tagF* had no effect on the phenotype of the Δ*pppA* mutant. Examination of the Δ*ppkA*Δ*tagF* double mutant showed that no Fha-mCherry foci were formed, consistent with the idea that PpkA-mediated phosphorylation is required for Fha focus formation. However in the Δ*ppkA*Δ*tagF* background, unlike Δ*ppkA*, foci of TssB-GFP were readily apparent (Fig 3D), in agreement with Hcp secretion and anti-bacterial activity being observed in this mutant. The TssB-GFP foci in Δ*ppkA*Δ*tagF* showed the same intensity distribution as in the wild type background, suggesting that the sheaths formed have similar properties (Fig 1D). Thus in a Δ*tagF* background, Fha phosphorylation and focus formation is not required for TssB foci or T6SS activity. However Fha itself is required, as no TssB-GFP focus formation was observed in a Δ*fha*Δ*tagF* mutant (Fig 3D).

**Fig 4:**
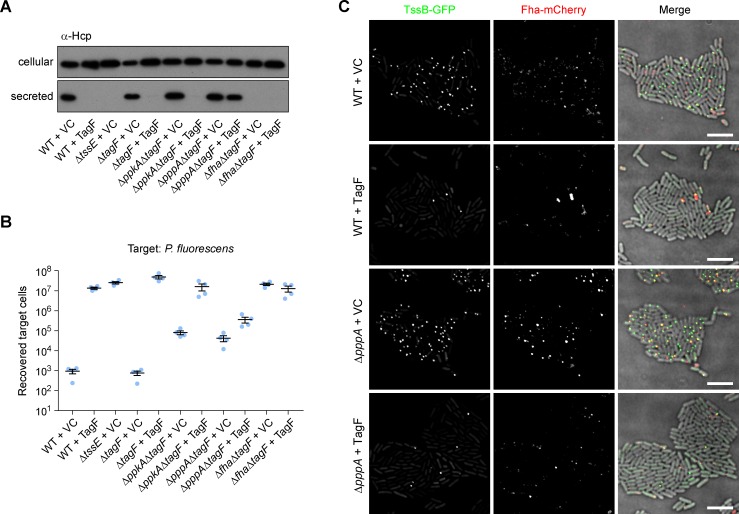
TagF is a negative regulator whose overexpression inhibits T6SS activity. (A) T6SS-dependent Hcp secretion as measured by immunoblot detection of Hcp in cellular and secreted fractions of wild type (WT) or mutant (Δ*tssE*, Δ*tagF*, Δ*ppkA*Δ*tagF*, Δ*pppA*Δ*tagF* and Δ*fha*Δ*tagF*) strains of *S*. *marcescens* Db10, carrying either the vector control plasmid (+VC, pSUPROM) or a plasmid directing the expression of *tagF in trans* (+TagF, pSC701). (B) T6SS-dependent anti-bacterial activity as determined by recovery of target organism *P*. *fluorescens* following co-culture with wild type or mutant strains of *S*. *marcescens* Db10, carrying vector control or *tagF* expression plasmid. Individual data points are overlaid with mean +/- SEM (n = 4). (C) Representative images of wild type and Δ*pppA* mutant strains of *S*. *marcescens* Db10 expressing TssB-GFP and Fha-mCherry fluorescent fusions and carrying either vector control or *tagF* expression plasmid. Panels show individual fluorescence channels (TssB-GFP, Fha-mCherry) and an overlay of the fluorescence channels and the DIC channel (Merge; GFP signal false-coloured green and mCherry red). Scale bar, 5 μm.

In an attempt to complement the phenotype of the Δ*tagF* mutant and also to establish the impact of TagF overexpression, *tagF* was introduced *in trans* in various genetic backgrounds. Overexpression of TagF in the wild type, Δ*tagF*, or Δ*ppkA*Δ*tagF* resulted in abolition of T6SS activity, consistent with a role as a negative regulator (Fig 4A and 4B). In contrast, some activity was retained upon overexpression of TagF in a Δ*pppA* background (Δ*pppA*Δ*tagF*). At the single cell level, TagF overexpression resulted in almost no TssB-GFP and Fha-mCherry foci in the wild type and only few foci in the Δ*pppA* mutant (Fig 4C). Taken all together, our data indicate that TagF negatively regulates assembly of a functional T6SS and that PpkA-mediated phosphorylation is required to overcome this repression.

### TagF represses T6SS membrane complex assembly

**Fig 5:**
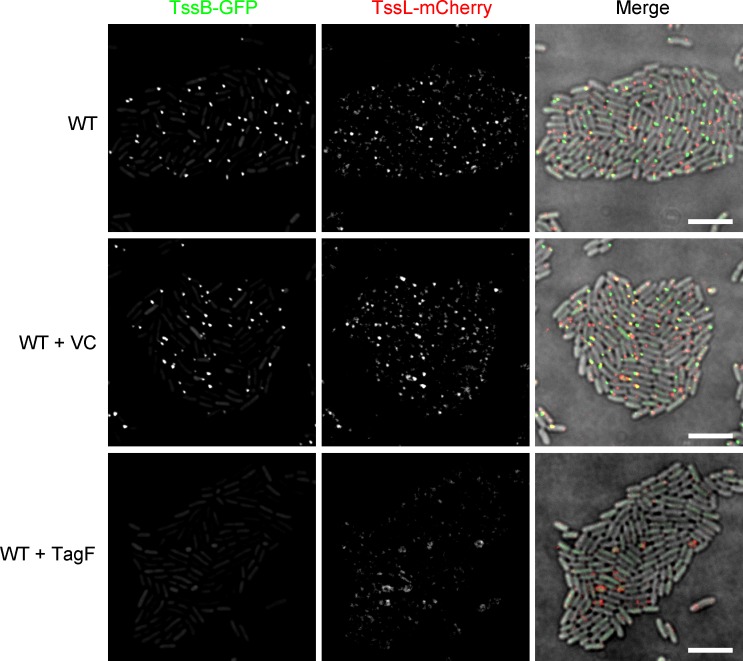
Overexpression of TagF inhibits formation of TssL-mCherry foci. Representative images of wild type (WT) *S*. *marcescens* Db10 expressing TssB-GFP and TssL-mCherry fluorescent fusions and carrying either the vector control plasmid (+VC, pSUPROM) or a plasmid directing the expression of *tagF in trans* (+ TagF, pSC701). Panels show individual fluorescence channels (TssB-GFP, TssL-mCherry) and an overlay of the fluorescence channels and the DIC channel (Merge; GFP signal false-coloured green and mCherry red). Scale bar, 5 µm.

Since TagF is encoded immediately downstream of TssM in *S*. *marcescens* and other T6SSs (Fig 3A, [21, 30]), we hypothesized that it might act upon the T6SS membrane complex (TssJLM). Formation of the membrane complex can be visualised by observing foci of the inner membrane proteins TssL or TssM [12, 17]. We observed that overexpression of TagF in a strain expressing TssB-GFP and TssL-mCherry from the chromosome [17] resulted in a loss of TssB-GFP foci, as expected. Importantly, TagF overexpression also caused a loss of the pronounced TssL-mCherry foci, leaving only more diffuse fluorescence (Fig 5), whilst total levels of TssL-mCherry protein were maintained (S5 Fig). Thus TagF is able to repress assembly of the T6SS membrane complex, which would subsequently prevent TssBC sheath assembly [12].

### RtkS activates the PpkA kinase in *S*. *marcescens*

**Fig 6:**
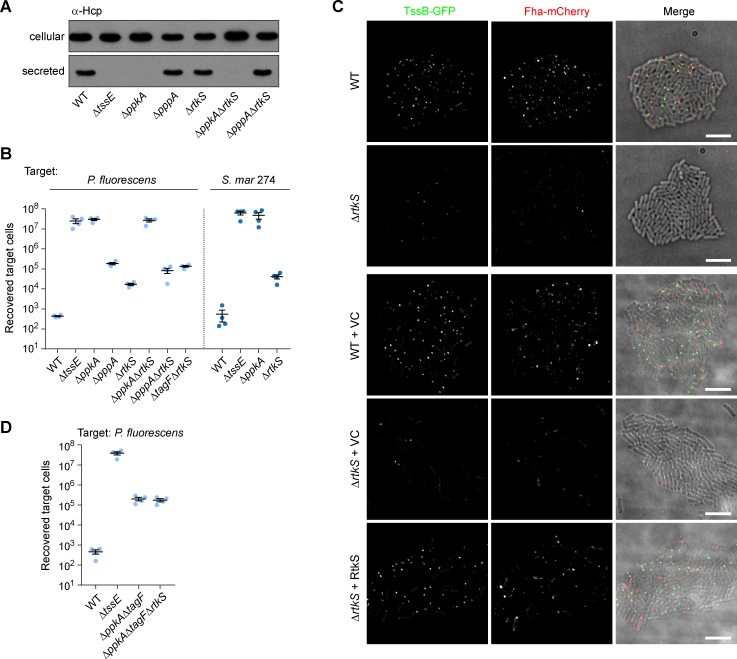
RtkS (SMDB11_2269) is a post-translational activator of T6SS activity. (A) T6SS-dependent Hcp secretion as measured by immunoblot detection of Hcp in cellular and secreted fractions of wild type (WT) or mutant (Δ*tssE*, Δ*ppkA*, Δ*pppA*, Δ*rtkS*, Δ*ppkA*Δ*rtkS* and Δ*pppA*Δ*rtkS*) strains of *S*. *marcescens* Db10. (B,D) T6SS-dependent anti-bacterial activity as determined by recovery of target organisms *P*. *fluorescens* or *S*. *marcescens* ATCC274 following co-culture with strains of *S*. *marcescens* Db10 (mutants as above, together with Δ*tagF*Δ*rtkS*, Δ*ppkA*Δ*tagF* and Δ*ppkA*Δ*tagF*Δ*rtkS*, as indicated). Individual data points are overlaid with mean +/- SEM. (C) Representative images of wild type (WT) and Δ*rtkS* mutant strains of *S*. *marcescens* Db10 expressing TssB-GFP and Fha-mCherry fluorescent fusions. In the lower three panels these strains are further carrying the vector control plasmid (+VC, pSUPROM) or a plasmid directing the expression of RtkS *in trans* (+ RtkS, pSC590). Panels show individual fluorescence channels (TssB-GFP, Fha-mCherry) and an overlay of the fluorescence channels and the DIC channel (Merge; GFP signal false-coloured green and mCherry red). Scale bar, 5 μm.

In *P*. *aeruginosa*, the kinase activity of PpkA is activated by a periplasmic sensing complex, comprising TagQRST and which responds to incoming T6SS attacks [18–21]. However in *S*. *marcescens*, this complex and the regulatory signal are not conserved [17, 27]. Aiming to identify the protein(s) acting upstream of PpkA in *S*. *marcescens* Db10, we noted a gene, *SMDB11_2269*, immediately downstream of *fha* and *pppA* (Fig 3A), encoding a protein of unknown function. Given the linkage with components of the TPP and the fact that it is predicted to be located in the periplasm (SignalP), we hypothesized that SMDB11_2269, subsequently named Regulator of T6SS kinase in *S**erratia* (RtkS), might represent an upstream regulator of PpkA. Under conditions of liquid growth, a mutant lacking RtkS (Δ*rtkS*) was able to secrete Hcp comparably to the wild type (Fig 6A). During co-culture with *P*. *fluorescens* or *S*. *marcescens* ATCC274, the Δ*rtkS* mutant showed a reduction in anti-bacterial activity compared with the parental strain, in both the wild type and the TssB-GFP Fha-mCherry background (Fig 6B, S1 Fig), implying that RtkS positively influences T6SS activity. No T6SS activity was detected in the Δ*ppkA*Δ*rtkS* double mutant, consistent with RtkS acting in the same pathway as PpkA. The Δ*pppA*Δ*rtkS* and Δ*tagF*Δ*rtkS* mutants showed a considerable reduction in efficiency of anti-bacterial activity, comparable with the Δ*pppA* mutant (Fig 6A and 6B). Observing TssB-GFP and Fha-mCherry in the Δ*rtkS* mutant revealed an obvious reduction in the number of bright TssB-GFP and Fha-mCherry foci, which could be restored by expression of RtkS *in trans* (Fig 6C). Complementation of the phenotype of the Δ*rtkS* mutant in the anti-bacterial activity assay could not be demonstrated, since overexpression of RtkS produced a similar reduction in efficiency as loss of the gene (S6 Fig). However when the Δ*rtkS* mutation was introduced into the Δ*ppkA*Δ*tagF* mutant, the genetic background in which the T6SS is functional in the absence of PpkA, loss of RtkS had no impact on anti-bacterial activity (Fig 6D). Firstly, this confirms that the Δ*rtkS* mutation has no secondary effects. More importantly, it shows that loss of RtkS has no impact in the absence of PpkA, implying that the only role of RtkS is in the TPP pathway.

**Fig 7:**
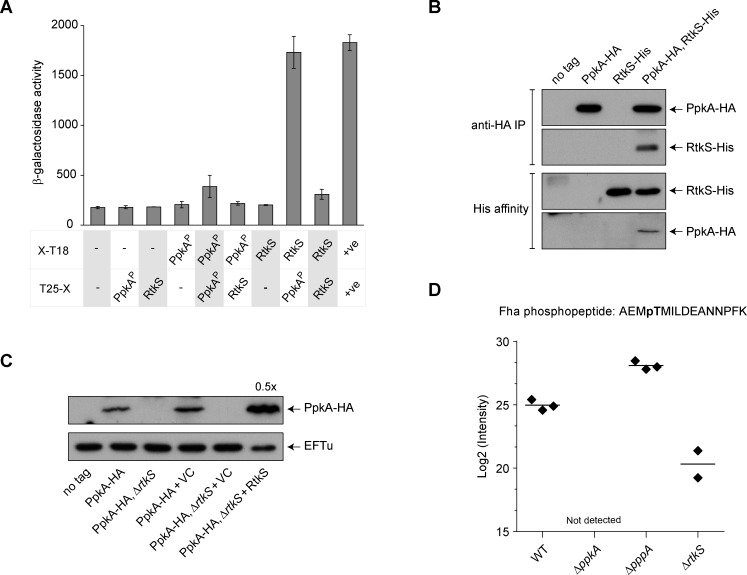
RtkS interacts with the periplasmic domain of the kinase PpkA, promoting its stability and consequent phosphorylation of Fha. (A) Bacterial two-hybrid assay to detect interactions between the periplasmic domain of PpkA (PpkA^P^; amino acids 363–482) and mature RtkS (RtkS; amino acids 20–328), each fused with T18 or T25 (PpkA^P^-T18, pSC593; T25-PpkA^P^, pSC594; RtkS-T18, pSC591; T25-RtkS, pSC592). Negative controls were provided by the empty vectors, pUT18 and pT25 (-), and a positive control by the self-interaction of TssK (+ve; TssK-T18, pSC048, and T25-TssK, pSC053). Shown is the β-galactosidase activity, expressed as Δ405/min/ml/OD_600_, of the reporter strain transformed with the combinations of plasmids indicated. Bars show mean +/- SEM (n = 3 independent transformations). (B) Co-purification of RtkS and PpkA under native conditions. Total membrane fractions of wild type *S*. *marcescens* Db10 (no tag) or strains expressing PpkA with a C-terminal HA tag (PpkA-HA), RtkS with a C-terminal His_6_ tag (RtkS-His), or both, from the normal chromosomal location were subjected to anti-HA immunoprecipitation (top panels) or His affinity purification using Ni^2+^-NTA (bottom panels). Bound proteins in each case were separated by SDS-PAGE and subjected to anti-HA immunoblot (to detect PpkA-HA) or anti-His_6_ immunoblot (to detect RtkS-His), as indicated. (C) Levels of PpkA in the presence or absence of RtkS were determined by immunoblot detection of PpkA-HA in an otherwise wild type background (PpkA-HA) or in the Δ*rtkS* mutant (PpkA-HA, Δ*rtkS*), and in strains carrying the vector control plasmid (+VC, pSUPROM) or a plasmid directing the expression of RtkS *in trans* (+ RtkS, pSC590). PpkA-HA levels were measured in total protein samples, with EFTu (bottom panel) representing a loading control and 0.5x the amount of total protein loaded in the right hand most lane compared with the other samples, as indicated. (D) Quantification of Fha phosphorylation by detemination of the levels of Fha phosphopeptide in otherwise wild type (WT) and mutant (Δ*ppkA*, Δ*pppA*, Δ*rtkS*) strains of *S*. *marcescens* Db10 expressing Fha-HA from the normal chromosomal location. Levels of phosphopeptide were determined by label-free mass spectrometry and individual data points are shown, with the mean indicated by a line. Levels of phosphopeptide were quantified in 3/3 replicates for WT and Δ*pppA*, and in 2/3 replicates for Δ*rtkS*. The phosphopeptide was not detected in the Δ*ppkA* mutant.

The observed reduction in assembled T6SSs and Fha foci was consistent with a role for RtkS in PpkA activation, so we investigated the potential link between RtkS and PpkA further. To test whether RtkS interacts with PpkA, we used the bacterial two-hybrid system [31]. This yielded a clear positive result, providing evidence for a direct interaction between RtkS and the periplasmic domain of PpkA from *S*. *marcescens* (Fig 7A). To confirm this interaction in the physiologically-relevant context, we generated strains expressing PpkA fused with a C-terminal haemagglutinin (HA) tag (PpkA-HA) and/or RtkS fused with a C-terminal hexahistidine tag (RtkS-His), with the fusion proteins being expressed from the normal chromosomal locations. Strains carrying either or both fusions exhibited normal T6SS-dependent anti-bacterial activity, confirming that the fusions did not impair function (S7 Fig, [32]). Using membrane fractions prepared from these strains, specific co-immunoprecipitation of RtkS-His with PpkA-HA, and, conversely, co-purification of PpkA-HA with RtkS-His could be observed (Fig 7B).

Given that RtkS had been shown to interact with PpkA, we investigated whether RtkS affected the stability of PpkA. By introducing the Δ*rtkS* mutation into the strain expressing PpkA-HA, it was observed that total protein levels of PpkA were greatly reduced by loss of RtkS (Fig 7C). This phenotype could be complemented by the expression of RtkS *in trans*, indeed excess RtkS resulted in levels of PpkA exceeding those in the wild type strain. The effect is specific to PpkA, since the cellular levels of other T6SS proteins, including Fha, were unaffected by loss of RtkS (Fig 6, S1 Fig). These data suggested that PpkA-dependent phosphorylation of Fha should be markedly reduced in the Δ*rtkS* mutant. Therefore, we measured Fha phosphorylation in the wild type, Δ*ppkA*, Δ*pppA* and Δ*rtkS* strains by a phosphoproteomic approach. Thr438 of Fha has been shown previously to be the sole target of PpkA in *S*. *marcescens* Db10 [27], therefore mass spectrometry was used to quantify the relative phosphorylation levels of Thr438 in each genetic background. Cells were harvested following growth on solid media, for comparability with the conditions used for anti-bacterial activity assays and single cell microscopy (ie where the T6SS is involved in productive cell-cell interactions). Levels of Fha phosphorylation in the wild type were ~12% of those in the Δ*pppA* mutant and the phosphopeptide was undetectable in the Δ*ppkA* kinase mutant, similar to our previous study where cells were grown in liquid cultures [27]. In the Δ*rtkS* mutant, phosphorylation of Fha was still detectable but was greatly reduced, to around 4% of wild type levels (Fig 7B). Thus RtkS is required for proper phosphorylation of Fha, consistent with a role as an upstream activator of PpkA which stabilises the kinase through a direct interaction.

## Discussion

Whilst anti-bacterial T6SSs are widespread and effective weapons during inter-bacterial competition, the broad strategy by which they are deployed can vary dramatically, even between similar systems. Some systems, such as *P*. *aeruginosa* H1-T6SS and EAEC SciX T6SS, are described as ‘defensive’ since they assemble and fire in response to an incoming attack from a neighbouring cell, whereas others are considered ‘offensive’ since they fire without any requirement for incoming attack or other cell contact-based signal, including the T6SSs of *V*. *cholerae* and *S*. *marcescens* [16–18]. The best characterised defensive system is *P*. *aeruginosa* H1-T6SS, where incoming attack is sensed by the TagQRST proteins, causing activation of the PpkA kinase, phosphorylation of Fha and assembly of an actively-firing T6SS at the site of the assault. This TPP regulatory system results in a single cell ‘dueling’ phenotype where neighbouring cells repeatedly assemble adjacent, retaliatory T6SS foci at the interface between them, and an inability of wild type *P*. *aeruginosa* to kill non-aggressive competitors such as *E*. *coli* K12 or secrete proteins in liquid media [18, 21]. On the other hand, the *S*. *marcescens* Db10 T6SS represents a typical example of an offensive system, killing non-attacking competitors efficiently and readily firing in liquid or in isolation from other cells [17, 26, 27]. Interestingly, however, the *P*. *aeruginosa* and *S*. *marcescens* T6SSs share a similar TPP pathway and TagF, and both require PpkA-mediated phosphorylation of Fha for T6SS activity, despite the opposite final regulatory outcome [21, 27]. This study has now demonstrated that the interaction of the TPP pathway with the core machinery is conserved between the offensive and defensive systems, whilst a novel input protein, RtkS, forms the top of the offensive pathway.

Using single-cell microscopy, we have shown that PpkA-mediated phosphorylation promotes formation of Fha foci in the offensive *S*. *marcescens* system, as also observed in the defensive *P*. *aeruginosa* system [20]. By simultaneously examining Fha-mCherry and TssB-GFP, we saw frequent co-occurrence of Fha foci with TssB sheath structures in the wild type background, implying that multiple copies of Fha associate with the T6SS machinery. TssB foci were not formed in the absence of PpkA or Fha. This, together with the fact that foci of Fha without associated TssB foci were readily identified, suggests that interaction of phosphorylated Fha with the T6SS basal complex precedes, and is required for, sheath assembly upon the basal complex (rather than, or in addition to, recruiting TssH as proposed initially [20]). TssB foci without obvious associated Fha foci were also observed. This may simply reflect the much lower intensities of Fha foci or may be as a result of a high frequency of sheath detachment from the baseplate following contraction [33]. Our data are consistent with observations in *P*. *aeruginosa*, where ClpV1(TssH)-GFP foci were co-located with Fha-mCherry foci (confirming Fha is associated with post-contraction machineries) but many other Fha foci were not associated with ClpV, implying that Fha is also associated with extended or pre-sheath machineries [20]. Quantification of TssB-GFP foci revealed a higher frequency of focus formation in a Δ*pppA* mutant, consistent with Fha phosphorylation promoting T6SS assembly. Surprisingly, however, the intensity of the TssB foci also increased significantly in the Δ*pppA* mutant. This implies an Fha-related difference in the structure or behaviour of the sheath itself.

We have previously noted that a Δ*pppA* mutant, whilst able to fire as efficently as the wild type, exhibits a significantly reduced efficiency of anti-bacterial activity [27]. This is consistent with the idea that PppA, perhaps by promoting partial or full disassembly of the basal machinery, facilitates spatial re-orientation of the T6SS between firing events [18]. This re-positioning would allow more than one neighbouring cell to be targeted by the same attacking cell. By comparing the temporal behaviour of TssB-GFP foci in wild type and Δ*pppA* strains, we confirmed that spatial relocation between firing events is reduced in the absence of PppA, with the T6SS frequently firing again in the same place in the Δ*pppA* mutant. Comparison of our findings with those of Basler *et al*., implies a conserved role for PppA-mediated dephosphorylation in spatial reorientation in both offensive and defensive T6SSs. In *P*. *aeruginosa*, there were many more (~10x) foci of ClpV1 (TssH) in a Δ*pppA* mutant compared with the wild type and these foci tended to remain localised to a single subcellular location [18]. In *S*. *marcescens*, the number of TssB foci is more comparable between the *pppA* mutant and the wild type (~1.5x), yet still the foci in the *pppA* mutant show a clear deficit in re-orientation. In both organisms, loss of PppA causes significant deficiency in anti-bacterial activity, demonstrating that ability to reorient is vital, whether the organism duels or simply attacks randomly.

The post-translational regulatory network of the T6SS in *S*. *marcescens* is further extended by the conserved negative regulator, TagF. As in *P*. *aeruginosa*, PpkA is no longer required for T6SS activity in the absence of TagF [21]. Moreover, the examination of single cells revealed that in the absence of TagF, formation of Fha foci is not required for sheath assembly or T6SS activity. Thus PpkA phosphorylation-dependent association of multiple copies of Fha with the T6SS, likely via oligomerisation, is required to overcome TagF-mediated repression but is not required for the basic firing function. Indeed recent work has indicated that Fha and TagF may interact directly [34]. However, even in the absence of TagF, Fha itself is required for sheath assembly and T6SS activity. Thus at least one copy of Fha is essential, fulfilling a second, phosphorylation-independent function in T6SS assembly. Such a function of Fha has been proposed [21] and is consistent with the presence of Fha in many organisms, like *V*. *cholerae*, which do not possess a TPP. It can be speculated that Fha, with this original function, was then adopted as a suitable target to allow integration of flexible modes of post-translational regulation upon T6SS activity. Importantly, we provide first evidence that TagF acts to prevent assembly of the membrane complex, which is the first step in assembly and firing of the T6SS. Mature membrane complexes allow docking of the cytoplasmic baseplate and form potential sites for assembly of the remainder of the T6SS [10, 12, 17]. Interaction of TagF with the membrane complex is not unexpected, given that *tagF* genes are tightly linked with *tssM* genes, and also that a hybrid PppA-TagF protein is found in the *Agrobacterium tumefaciens* T6SS, in which the target of PpkA-mediated phosphorylation is TssL [30]. Whether the action of TagF is as simple as physically occluding assembly of the mature membrane complex from smaller TssJLM or Tss(JLM)_2_ units [35], or if it has a more subtle impact on basal complex assembly, remains to be determined.

Having shown that the downstream interaction of the TPP and TagF regulatory system with the core machinery is conserved between the offensive *S*. *marcescens* and the defensive *P*. *aeruginosa* T6SSs, we considered the upstream input of the TPP pathway. In *P*. *aeruginosa*, the trans-membrane TagQST complex detects membrane breach caused by incoming T6SS attacks, causing the outer membrane-associated periplasmic protein TagR to activate PpkA, probably by direct interaction of TagR with the periplasmic domain of PpkA triggering dimerisation and autophosphorylation of the kinase [18–21]. In contrast, we have shown that a new input protein, RtkS, positively regulates PpkA-mediated phosphorylation in *S*. *marcescens*. RtkS interacts directly with the periplasmic domain of *S*. *marcescens* PpkA, which is distinct from the periplasmic domain of *P*. *aeruginosa* PpkA [27], and is required for the stability and therefore the kinase activity of PpkA. We propose that this stabilising interaction may be modulated in response to T6SS-activating or -inactivating signals, for example via a small molecule ligand for RtkS. Alternatively, it is also possible that RtkS stabilises PpkA in all situations but can activate the PpkA kinase activity in response to a specific signal. A periplasmic location for RtkS is evidenced by its possession of a strong consensus N-terminal signal peptide and the fact that it requires modification by the periplasmic enzyme DsbA for stability [32]. Homologues of RtkS are present not only in other strains of *S*. *marcescens*, but also in related T6SSs in other species, including *Enterobacter cloacae*, *Pantoea* sp. and *Erwinia amylovora*, and we speculate that the presence of an RtkS homologue may define an offensive system.

The signal detected by RtkS is currently unknown. RtkS is not related to any known signalling or small molecule binding proteins, and no predicted structural homologies are readily identifiable. Interestingly, the loss of RtkS appeared to have a somewhat variable impact on T6SS activity, depending on which assay was utilised. This might partly reflect differences in the growth conditions, for example microscopy analyses requiring use of a minimal media, and may suggest the existence of an RtkS-independent means of PpkA activation in certain situations. Another potential layer of control of T6SS activity in *S*. *marcescens* in response to competitors is at the transcript level. It has been reported that the RcsAB two-component system regulates expression of the *S*. *marcescens* RM66262 T6SS at a transcriptional level, and may upregulate the expression of the T6SS in response to effectors delivered by competitor cells [36]. This system may offer some parallels with the ‘PARA’ response in *P*. *aeruginosa* [37]. Lazarro *et al*. further suggested that the outcome of RcsAB-mediated transcriptional regulation is that *S*. *marcescens* can intoxicate both wild type competitor *S*. *marcescens* and T6SS-lacking *E*. *coli*, but cannot intoxicate T6SS-inactive mutants of *S*. *marcescens* [36]. However, we do not believe that this conclusion can be generally applied to all *S*. *marcescens*, having observed that Db10 can intoxicate T6SS-inactive mutants of itself or of other strains of *S*. *marcescens* as efficiently as T6SS-active strains [17, 25, 38].

**Fig 8:**
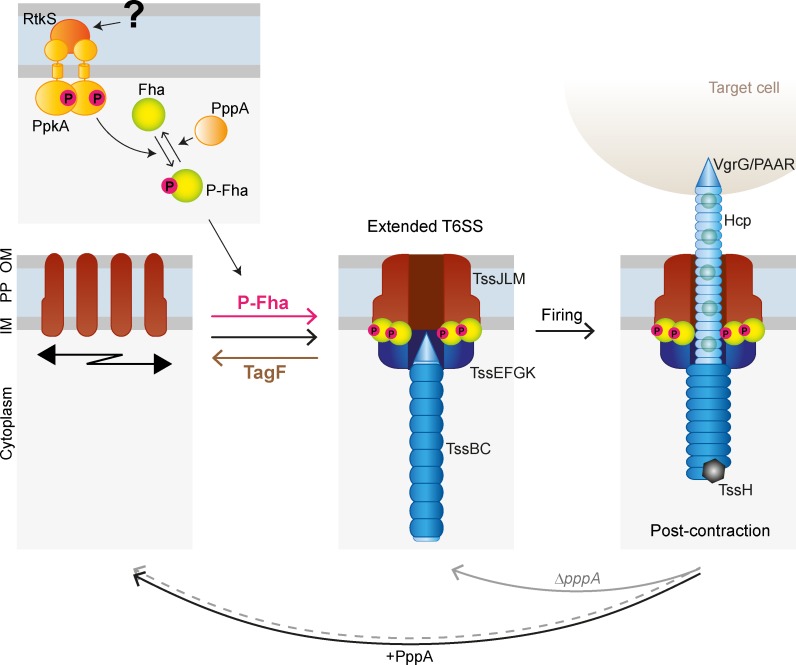
Schematic model for post-translational regulation of T6SS firing in *S*. *marcescens*. Prior to assembly of the T6SS basal complex (membrane complex, TssJLM, plus baseplate complex, TssEGFK-VgrG), the membrane complex components are free to move around the cell. TagF inhibits assembly of the mature membrane complex which represents the site of baseplate docking. In response to an unknown signal, RtkS interacts with the periplasmic domain of PpkA, leading to PpkA autophosphorylation and phosphorylation of Fha, which is antagonised by PppA. Phosphorylated Fha multimerises and associates with the T6SS, overcoming TagF-mediated repression of basal complex formation. Next, the inner Hcp tube and surrounding TssBC sheath assemble on VgrG and TssE, respectively, and extend into the cytoplasm. This ‘extended’ T6SS is primed and ready to fire. During the firing event, the TssBC sheath contracts, propelling the Hcp-VgrG-PAAR puncturing structure out of the cell through the membrane complex and into a target cell. Effectors decorating the Hcp-VgrG-PAAR structure are released into a target cell, or else to the medium if no target is present. TssH then binds the contracted TssBC sheath and initiates depolymerisation, the basal complex disassembles and PppA dephosphorylates Fha. For a new round of firing to occur, in a wild type cell, TagF-mediated repression must again be overcome by phosphorylation of Fha, allowing time for the T6SS components to move and reassemble in a new spatial location. In the absence of PppA, Fha remains fully phosphorylated, leading to a tendency for the T6SS to immediately reassemble in the same place. When PpkA and TagF are both absent, the intrinsic ability of the system to assemble is observed but anti-bacterial activity is less efficient than with proper post-translational activation in place. IM, inner membrane; PP, periplasm; OM, outer membrane.

All together, our data allow us to propose a model for post-translational regulation of T6SS firing in *S*. *marcescens*, described in Fig 8. Prior to assembly, the T6SS membrane complex components move freely around the cell surface and TagF inhibits formation of the mature membrane complex. Activation of the TPP by RtkS leads to phosphorylation of Fha, causing multiple Fha proteins to associate with the T6SS. This overcomes TagF-mediated repression and promotes basal complex formation, allowing the entire T6SS structure, including the extended TssBC sheath, to form and fire. Following firing and effector delivery, TssH depolymerises the contracted sheath, either before or concurrent with dissociation of the basal complex and dephosphorylation of Fha by PppA. For a new round of firing to occur, TagF-mediated repression must again be overcome by phosphorylation of Fha. The time for RtkS to sense the signal, activate PpkA and overcome PppA activity in order to achieve a threshold level of Fha phosphorylation provides an interval allowing the T6SS machinery to move and reassemble in a new spatial location. Thus the opposing activities of TagF and the TPP act as a built-in timer to permit spatial re-location of the T6SS and maximum efficiency of anti-bacterial activity. This timer could further be modulated by signal availability and any RtkS-independent input. In a Δ*pppA* mutant, Fha remains fully phosphorylated, leading to a tendency for the T6SS to immediately reassemble in the same place. When PpkA and TagF are both absent, the intrinsic ability of the T6SS to assemble is observed but anti-bacterial activity is less efficient. This may explain why the *V*. *cholerae* T6SS, an offensive T6SS lacking TPP and TagF, needs to fire more frequently [15]. In *P*. *aeruginosa*, it is likely that the same timer function operates but spatial re-orientation is further refined by the activating signal being specifically localised at the site of incoming attack. Hence the same basic regulatory function of the TPP-TagF module is applied in both offensive and defensive T6SSs.

In conclusion, we have used a combination of genetic and single cell analysis to provide an integrated model for post-translational regulation of T6SS firing. The TPP-TagF regulatory system is flexible, accommodating different inputs to provide distinct outcomes, and increases efficiency of anti-bacterial activity by maximising effective spatial targeting of competitors.

## Methods

### Bacterial strains, plasmids, and culture conditions

The strains and plasmids used in this study are described in S1 Table. Strains of *S*. *marcescens* Db10 carrying in-frame deletions or encoding protein fusions with GFP or mCherry at the normal chromosomal location were generated by allelic exchange using the pKNG101 suicide vector as described previously [17, 26]. For Fha-mCherry reporter strains, the wild type allele was replaced with one encoding a fusion of mCherry to the C-terminus of the Fha protein, separated by a a GAGAPVAT linker and with a second copy of the final nine amino acids of native Fha added to the C-terminus of mCherry in order to preserve expression of the downstream gene. The mCherry gene was amplified from pmCherry-N1 (Clontech). Plasmids for constitutive expression of genes *in trans* were derived from pSUPROM. Details of plasmid construction and primer sequences are given in S2 Table. The genome sequence of *S*. *marcescens* Db11 is used for *S*. *marcescens* Db10 since they differ only through a point mutation in *rpsL* in Db11 [39]. Strains of *S*. *marcescens* were grown at 30°C in LB (10 g/l tryptone, 5 g/l yeast extract, 10 g/l NaCl, with 1.2 g/l agar for solid media) or minimal glucose medium (40 mM K_2_HPO_4_, 15 mM KH_2_PO_4_, 0.1% (w/v) (NH_4_)_2_SO_4_, 0.4 mM MgSO_4_, 0.2% (w/v) glucose). Strains of *E*. *coli* were grown in LB at 37°C unless stated otherwise. When required, cultures were supplemented with antibiotics: kanamycin (Kn) 100 µg/ml; streptomycin (Sm) 100 µg/ml, chloramphenicol (Cm) 25 µg/ml or ampicillin (Ap) 100 µg/ml.

### Immunoblotting and measurement of T6SS activity

Anti-Hcp1 immunoblots were performed on total cellular and supernatant samples from cultures grown for 7 h in LB as described previously [26]. Total cellular fractions from strains expressing TssB-GFP, Fha-mCherry, TssL-mCherry or PpkA-HA fusion proteins, grown for 7 h in LB, were probed using primary antibodies against GFP (Roche, 1:10,000), mCherry (Abcam, 1:10,000) or HA tag (MRC PPU Reagents, University of Dundee, 1:6,000) and peroxidase-conjugated anti-mouse secondary antibody (BioRad, 1:10,000).

T6SS-mediated anti-bacterial activity was measured using a co-culture assay based on that described previously [26]. Briefly, the attacker strain of *S*. *marcescens* Db10 and the target strain, both at OD_600_ 0.5, were mixed at an initial attacker:target ratio of 1:1 and co-cultured on solid LB at 30°for 4 h. Surviving target cells were subsequently enumerated by serial dilution and viable counts on streptomycin-supplemented media. *P*. *fluorescens* KT02 was normally used as target, except when kanamycin selection of plasmids in the attacking strains was required, when KT03 was used, or when *S*. *marcescens* ATCC274 was required as target, when SJC17 was used (S1 Table).

### Fluorescence microscopy

For microscopy analysis, overnight cultures were diluted to an OD_600_ of 0.025 in 25 ml minimal glucose media and incubated for 4–5 h at 30°C with shaking. From this, 1.5 µl of bacterial culture was placed on a microscope slide layered with a pad of minimal glucose medium solidified by the addition of 1.5% UltraPure agarose (Invitrogen) and sealed with 1.5 thickness coverslips (VWR) attached to the microscope slide with a GeneFrame (Thermo). The slides were then allowed to equilibrate in the microscope chamber, pre-heated to 30°C, for minimum of 10 min prior to imaging.

All images were acquired using a DeltaVision Elite widefield microscope (GE Healthcare Life Sciences) fitted with an Olympus 100x 1.4 NA lens and a CoolSnap HQ2 camera (Photometrics), with differential interference contrast (DIC) and fluorescence optics. Datasets of 512 × 512 pixels with up to 11 Z-sections spaced by 0.25 µm were collected. Images were acquired with an LED transmitted light source (Lumencor solid state system). Unless stated otherwise, GFP and mCherry were detected using a GFP filter set (Ex 480/25 nm, Em 525/30 nm), exposure time 150 ms with 50% transmission, and a mCherry filter set (Ex 575/25, Em 628/40), exposure time 200 ms with 100% transmission. DIC images were acquired at 50% transmission and exposure time of 80 ms. Following the acquisition, images were deconvolved using softWoRx and processed and stored using OMERO (http://openmicroscopy.org) [40]. Images were collected on three independent occasions for each strain analysed, with at least 10 (and typically 15) fields of view on each occasion. Representative examples were used for figure preparation with the aid of OMERO.figure (http://figure.openmicroscopy.org). All data was acquired within the dynamic range of the camera and all quantitation was performed on the raw images. Images presented in figures in this manuscript have been adjusted to aid clarity, with all comparable images (e.g. the same fusion in different mutant backgrounds) treated identically.

### Quantitative image analysis

For signal intensity analysis, the images were prepared using the OMERO.insight image manager. Regions of interest (ROIs) were drawn around visible groups of cells and propagated in the Z dimension to encompass the thickness of whole cells. A minimum of 32 images per strain was analysed using OMERO.mtools software (http://www.openmicroscopy.org/site/products/partner/omero.mtools). For detection of fluorescent foci, fluorescence images stored in OMERO were segmented, in other words each pixel was assigned to white (‘focus’) or black (‘not focus’), by a thresholding approach. In each ROI, foci were defined by having signal intensity greater than 2.2 standard deviations from the mean signal intensity throughout the whole ROI, and also being larger than six pixels. The resulting binary masks were stored in OMERO for further analysis. The signal intensity of objects (foci) from the segmented image masks was measured and output (as mean intensity of all pixels in the object) to an Excel spreadsheet. The background signal around each segmented object with annulus of 1 pixel was also measured and was subsequently subtracted from the measured signal intensities.

### Analysis of TssB-GFP dynamics

To analyse the dynamics of assembly and disassembly of TssB-GFP fusion protein, a time-lapse microscopy experiment was performed using the DeltaVision Elite microscope described above. Images were acquired in the GFP and DIC channels every 90 s over the course of 30 min. The GFP exposure time was 80 ms with 100% transmission, the DIC exposure time was 50 ms with 50% transmission. Eleven Z-sections were acquired for each image with Z-step of 200 nm. The resulting images were deconvolved in softWoRx and stored in OMERO. To generate temporal projection images, the original images were taken for analysis using Fiji [41] and the highest contrast Z-section of the GFP channel was isolated. Stage drift was corrected using the StackReg plugin [42] with the ‘Rigid Body’ setting. The rendering of all images was set to min 400, max 5500, a setting where all foci were visible throughout the time lapse. The ‘temporal projection’ to visualise all of the foci through the time of the experiment was generated using the Temporal-Color Code macro (http://imagej.net/Temporal-Color_Code) with the lookup table presented in Fig 2. The resulting projection was overlaid with the DIC image to reconstruct the multichannel image. The corresponding time-lapse movies were generated by cropping the original composite image using Fiji.

### Bacterial two-hybrid analysis

Bacterial two-hybrid analyses were performed following established protocols [31, 43]. *E*. *coli* MG1655 Δ*cyaA* was co-transformed with combinations of a pUT18-based and a pT25-based plasmid and the color of the resulting transformants scored on MacConkey media with Ap, Cm and 0.2% maltose (positive result is red). For quantitative measurement of the interaction, β-galactosidase assays were performed as described [26] on double-transformed MG1655 Δ*cyaA* grown at 30°C in LB and permeabilized with toluene. Replicate assays were performed on three independent transformants.

### Co-purification of PpkA-HA and RtkS-His

*S*. *marcescens* strains harbouring chromosomally encoded PpkA-HA and/or RtkS-His_6_ fusion proteins were grown for 5 h in LB. Cells were recovered by centrifugation (4000 g, 30 min), washed in 20 mM Tris-HCl pH 7.8, resuspended in 20 ml of Tris-HCl pH 7.8 and lysed using an EmulsiFlex-C3 homogenizer (Avestin). Cell debris were removed by centrifugation (14.000 g, 45 min, 4°C) and 1 ml of the lysate (corresponding to 50 ml of the original culture) was subjected to ultracentrifugation (200.000 g, 30 min, 4°C). Following this step, the pellet representing the total membrane fraction was resuspended in 800 μl of 20 mM Tris-HCl pH 7.8, and n-dodecyl-β-d-maltoside and Triton X-100 were added to a final concentration of 2%. Membrane fractions were transferred into tubes containing 50 μl of pre-washed (3x) magnetic α-HA beads (NEB) or magnetic Ni^2+^-NTA beads (Qiagen) and incubated for 2 h at 4°C, 20 rpm. The beads were then washed with 4 x 1 ml of wash buffer (20 mM Tris-HCl pH 7.8, 100 mM NaCl. 0.1% Triton X-100) and bound proteins were eluted by addition of 40 μl of 2x SDS-PAGE sample buffer [26].

### Affinity isolation of HA-tagged Fha protein

Three biological replicates of *S*. *marcescens* Db10 harbouring a chromosomally encoded Fha-HA fusion protein in an otherwise wild type background, or in Δ*ppkA*, Δ*pppA* or Δ*SMDB11_2269* mutant backgrounds, were grown overnight at 30°C in LB. Cultures were normalised to an OD_600_ of 0.5 and 70 x 25 μl aliquots were spotted onto the surface of LB agar plates, followed by incubation for 5 h at 30°C. Cells from the 70 individual culture spots were recovered from the surface of the agar plate, pooled by resuspending together in 10 ml of LB, harvested by centrifugation at 16000 x *g* at 4°C for 20 min and washed with 10 ml of 1xPBS. After the wash, cells were resuspended in 1ml of lysis buffer (1xPBS pH 7.4, 1% (v/v) Triton X-100, phosphatase inhibitors [1.15 mm Na_2_MoO_4_, 1 mm Na_3_VO_4_, 4 mm Sodium tartrate, 5 mm glycerol 3-phosphate] and 1 tablet of complete, Mini, EDTA-free Protease Inhibitor Mixture [Roche]). Cells were disrupted by 6 cycles of sonication (20% amplitude, 15 s pulse and 30 s pause) on an ice-ethanol slush. Lysates were subjected to two centrifugation steps, each 20 min at 16000 x *g* at 4°C, to remove cell debris. Cleared cell extract was added to 30 μl anti-HA agarose conjugate (Sigma), previously washed x5 with 1 ml PBS pH 7.4, 0.2% (v/v) Triton X-100, and incubated for 1 h at 4°C on an orbital shaker. Beads were then washed x5 with 1 ml PBS pH 7.4, 0.2% (v/v) Triton X-100, 1:100 phosphatase inhibitor. Bound proteins were separated on NuPAGE 4–12% bis-tris acrylamide gels (Invitrogen) until stacked in a single band, stained with InstantBlue (Expedeon), cut from the gel, reduced (10 mM TCEP), alkylated (50 mM Iodoacetamide), digested with trypsin protease MS Grade (Pierce) and subjected to mass spectrometry analysis for identification.

### Mass spectrometry analysis of Fha phosphorylation

Peptide samples were separated on an ultimate 3000 Rapid Separation LC Systems chromatography (Thermo Scientific) with a C18 PepMap, serving as a trapping column (2 cm x 100 μm ID, PepMap C18, 5 μm particles, 100 Å pore size) followed by a 50 cm EASY-Spray column (50 cm x 75 μm ID, PepMap C18, 2 μm particles, 100 Å pore size)(Thermo Scientific) with a linear gradient of 2.4–20% (ACN, 0.1% FA) over 115 min followed by a step from 20–28% ACN, 0.1% FA over 30 min at 300 nL/min. Mass spectrometric identification was performed on an Orbitrap Fusion Tribrid mass spectrometer (Thermo Scientific) operated in “Top Speed” data dependent mode, operated in positive ion mode. FullScan spectra were acquired in a range from 400 m/z to 1600 m/z, at a resolution of 120 000 (at 200 m/z), with an automated gain control (AGC) of 300,000 and a maximum injection time of 50 ms. Charge state screening was enabled to exclude precursors with a charge state of 1. The intensity threshold for a MS/MS fragmentation was set to 10e4 counts. The most intense precursor ions were isolated with a quadrupole mass filter width of 1.6 m/z and HCD fragmentation was performed with one-step collision energy of 35% and activation Q of 0.25. MS/MS fragments ions were analysed in the segmented linear ion trap with a normal scan range, in a rapid mode. The detection of MS/MS fragments was set up as the “Universal Method”, using a maximum injection time of 300 ms and a maximum AGC of 2,000 ions.

Mass spectrometric raw data files of three biological replicates of each strain were searched against a combined database of *S*. *marcescens* Db11 containing 4,720 sequences and a list of common contaminants. Protein identification and label-free quantification were performed using MaxQuant Version 1.5.1.7 [44] with the following parameters: stable modification carbamidomethyl (C); variable modifications oxidation (M), acetylation (protein N terminus), deamidation (NQ) and phosphorylation (STY). Mass accuracy was set to 4.5 ppm for precursor ions and 0.5 Da for ion trap MS/MS data. Identifications were filtered at a 1% false-discovery rate (FDR) at the protein level. Label-free quantification of identified proteins referred to razor and unique peptides, and required a minimum ratio count of 2. Normalized ratios of proteins and identified phosphopeptides were extracted for each condition and used for downstream analyses.

## Supporting information

S1 Fig Confirmation of integrity and functionality of fluorescent fusion strains. (A) Immunoblot detection of cellular and secreted Hcp levels in wild type *S*. *marcescens* Db10 and derivatives expressing TssB-GFP, Fha-mCherry or both fusions, in wild type, Δ*ppkA*, Δ*pppA*, Δ*tagF*, Δ*ppkA*Δ*tagF*, Δ*pppA*Δ*tagF*, Δ*fha*Δ*tagF* and ΔSMDB11_*2269* mutant backgounds. (B) T6SS-dependent anti-bacterial activity of the same strains against *P*. *fluorescens*. The blots and co-culture data in (A) and (B) are from the same experiment as depicted in panels A and B in Fig 1, but are here shown in their entirety, with the fluorescent fusion strains from the whole study all together. (C) Immunoblot detection of TssB-GFP (left) and Fha-mCherry (right) in whole cell extracts of *S*. *marcescens* Db10 expressing the fusion proteins in the wild type and mutant backgrounds indicated.
(TIF)

S2 Fig Restoration of focus formation by Fha-mCherry upon complementation of the Δ*ppkA* mutation. (A) Representative fluorescence images of wild type *S*. *marcescens* Db10 (WT), the otherwise wild type derivative expressing the single Fha-mCherry fusion, and the Δ*ppkA* mutant with Fha-mCherry. The low level background signal visible in the WT panel is due to autofluorescent particles in the agarose pads. (B) Representative fluorescence images of wild type or Δ*ppkA* expressing TssB-GFP and Fha-mCherry and carrying either the vector control plasmid (VC, pSUPROM) or a plasmid directing the expression of PpkA *in trans* (+PpkA, pSC812). Left (and middle) panels show individual fluorescence channels (Fha-mCherry, TssB-GFP) and the right shows an overlay of the fluorescence channel(s) and the DIC channel (Merge; mCherry signal false-coloured red and GFP green); scale bars, 5 μm.
(TIF)

S3 Fig Additional examples of time-lapse imaging of TssB-GFP localisation in wild type and Δ*pppA* strains of *S. marcescens* Db10. Superimposed frames from a 30 min time-lapse experiment with image acquisition every 90 s, with each frame colour coded by time as indicated. Centre panels, colour coded fluorescence images of the microcolony; left panels, magnification of the region indicated by the white box; right panels, corresponding DIC image.
(TIF)

S4 Fig Full time-lapse sequences of TssB-GFP localisation within cells. Individual frames showing the whole microcolony for the time-lapse sequences in Fig 2, presented as overlaid images of the TssB-GFP and DIC channels. GFP signal is false-coloured in green and the acquisition time in seconds is indicated. Scale bar, 5 μm.
(TIF)

S5 Fig Total protein levels of TssL-mCherry and TssB-GFP on TagF overexpression. Immunoblot detection of TssL-mCherry, TssB-GFP and control housekeeping protein EFTu in whole cell extracts of wild type *S*. *marcescens* Db10 (WT) or the strain expressing TssL-mCherry and TssB-GFP, carrying either the vector control plasmid (+VC, pSUPROM) or a plasmid directing the expression of *tagF in trans* (+TagF, pSC701).
(TIF)

S6 Fig Complementation of the Δ*rtkS* mutant by expression of RtkS *in trans*. T6SS-dependent anti-bacterial activity as determined by recovery of target organism *P*. *fluorescens* following co-culture with wild type (WT) or mutant (Δ*tssE*, Δ*rtkS*, Δ*ppkA* and Δ*pppA*) strains of *S*. *marcescens* Db10, carrying either the vector control plasmid (+VC, pSUPROM) or a plasmid directing the expression of *rtkS in trans* (+ RtkS, pSC590). Individual data points are overlaid with mean +/- SEM (n = 4).
(TIF)

S7 Fig Chromosomally encoded PpkA-HA and RtkS-His fusions retain wild type functionality. T6SS-dependent anti-bacterial activity as determined by recovery of target organism *P*. *fluorescens* following co-culture with wild type (WT) or mutant (Δ*tssE*, Δ*ppkA*, Δ*rtkS*, PpkA-HA, PpkA-HA Δ*rtkS* and PpkA-HA RtkS-His) strains of *S*. *marcescens* Db10. Individual data points are overlaid with mean +/- SEM (n = 4).
(TIF)

S1 Movie Time-lapse imaging of TssB-GFP in wild type *S. marcescens* Db10. Four examples from timelapse experiments presented in S3 Fig.
(AVI)

S2 Movie Time-lapse imaging of TssB-GFP in Δ*pppA* of *S. marcescens* Db10. Four examples from timelapse experiments presented in S3 Fig.
(AVI)

S1 Table Bacterial strains and plasmids. (PDF)

S2 Table Oligonucleotide primers for plasmid construction. (PDF)
