# Supplementary figures and images for: Killing with proficiency: Integrated post-translational regulation of an offensive Type VI secretion system
Source: PLoS Pathog. 2018 Jul 27;14(7):e1007230. doi: 10.1371/journal.ppat.1007230 (PMC6082577; doi:10.1371/journal.ppat.1007230)

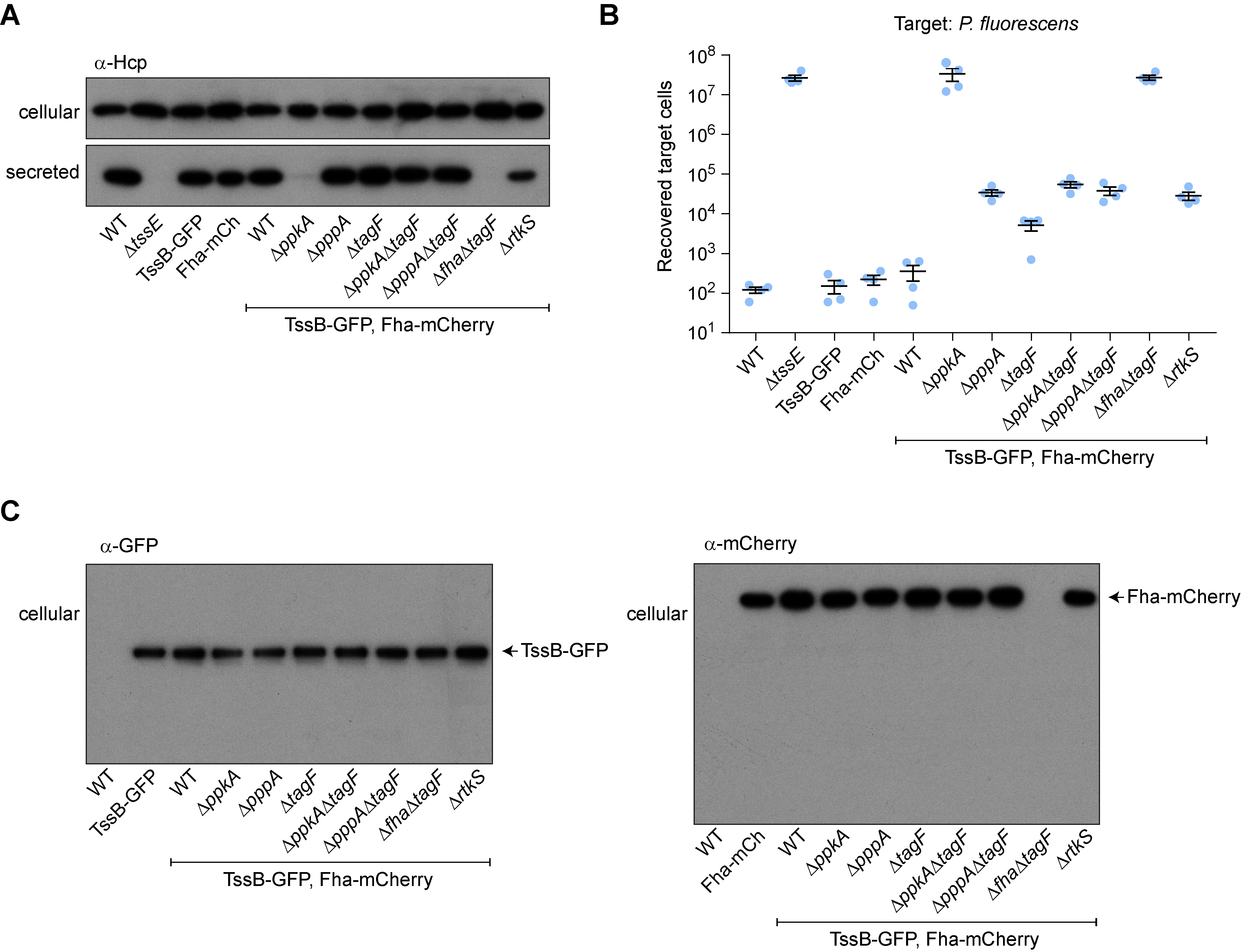

Supplement: S1 Fig — (A) Immunoblot detection of cellular and secreted Hcp levels in wild type S. marcescens Db10 and derivatives expressing TssB-GFP, Fha-mCherry or both fusions, in wild type, ΔppkA, ΔpppA, ΔtagF, ΔppkAΔtagF, ΔpppAΔtagF, ΔfhaΔtagF and ΔSMDB11_2269 mutant backgounds. (B) T6SS-dependent anti-bacterial activity of the same strains against P. fluorescens. The blots and co-culture data in (A) and (B) are from the same experiment as depicted in panels A and B in Fig 1, but are here shown in their entirety, with the fluorescent fusion strains from the whole study all together. (C) Immunoblot detection of TssB-GFP (left) and Fha-mCherry (right) in whole cell extracts of S. marcescens Db10 expressing the fusion proteins in the wild type and mutant backgrounds indicated. (TIF) [file ppat.1007230.s001.tif]

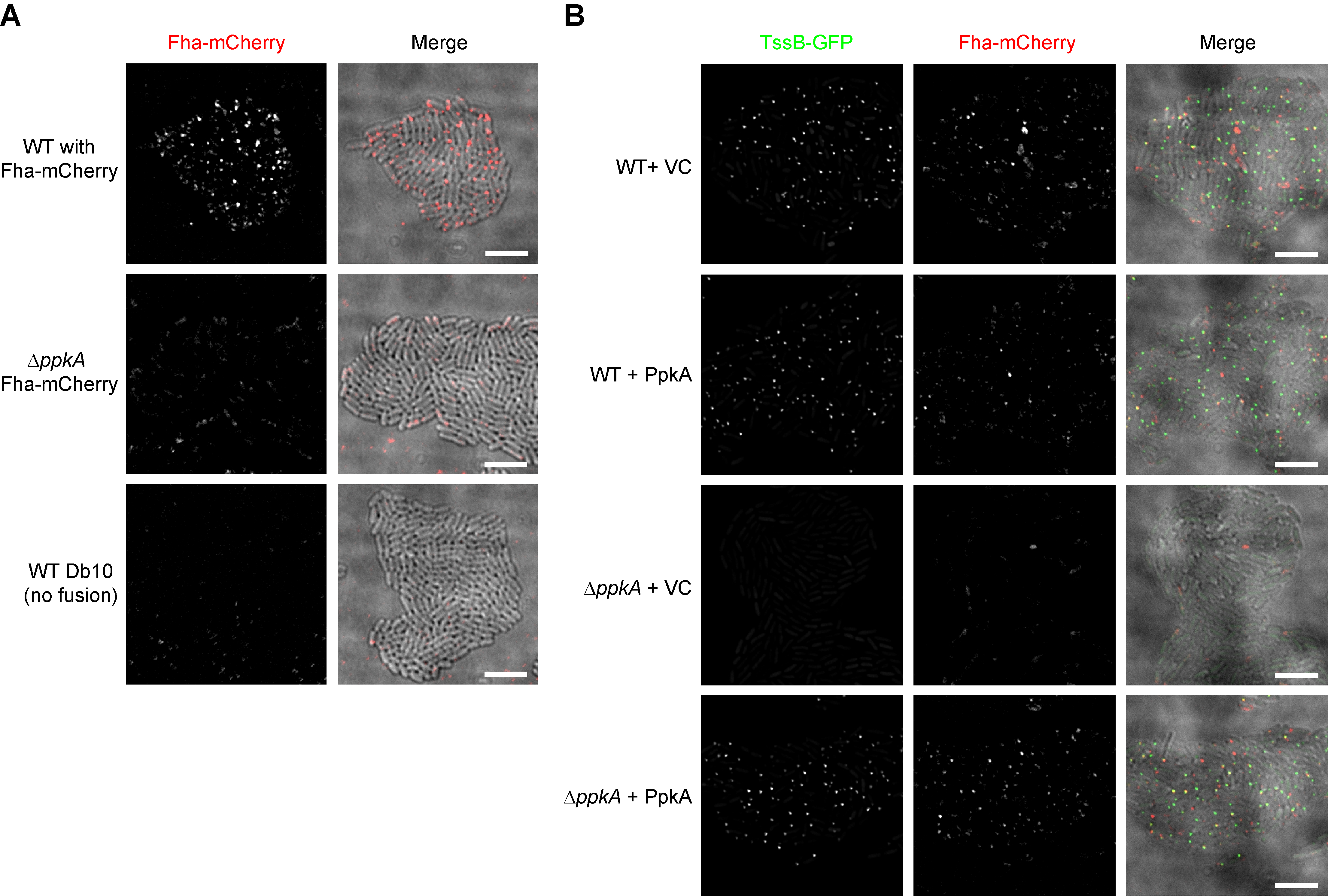

Supplement: S2 Fig — (A) Representative fluorescence images of wild type S. marcescens Db10 (WT), the otherwise wild type derivative expressing the single Fha-mCherry fusion, and the ΔppkA mutant with Fha-mCherry. The low level background signal visible in the WT panel is due to autofluorescent particles in the agarose pads. (B) Representative fluorescence images of wild type or ΔppkA expressing TssB-GFP and Fha-mCherry and carrying either the vector control plasmid (VC, pSUPROM) or a plasmid directing the expression of PpkA in trans (+PpkA, pSC812). Left (and middle) panels show individual fluorescence channels (Fha-mCherry, TssB-GFP) and the right shows an overlay of the fluorescence channel(s) and the DIC channel (Merge; mCherry signal false-coloured red and GFP green); scale bars, 5 μm. (TIF) [file ppat.1007230.s002.tif]

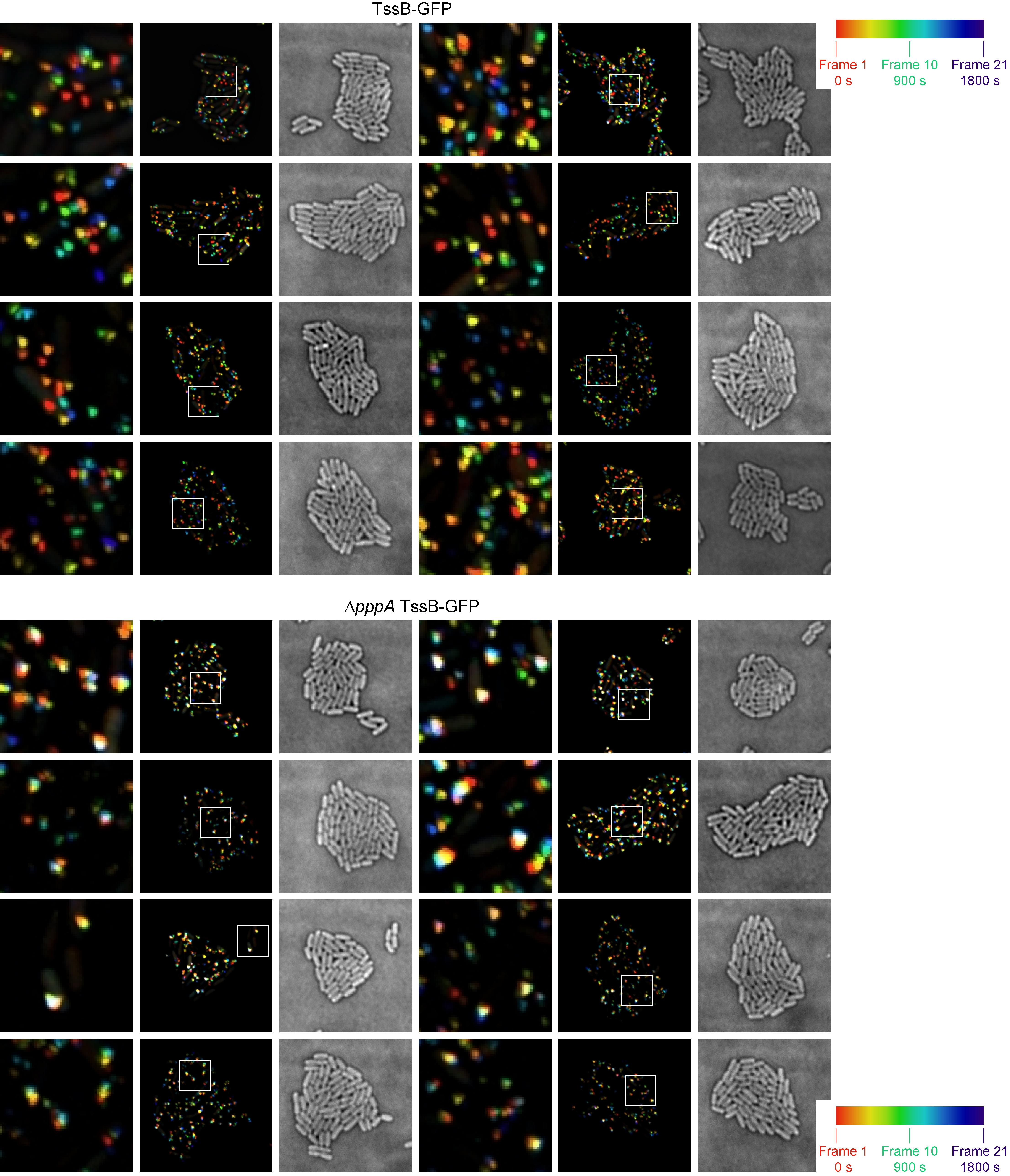

Supplement: S3 Fig — Superimposed frames from a 30 min time-lapse experiment with image acquisition every 90 s, with each frame colour coded by time as indicated. Centre panels, colour coded fluorescence images of the microcolony; left panels, magnification of the region indicated by the white box; right panels, corresponding DIC image. (TIF) [file ppat.1007230.s003.tif]

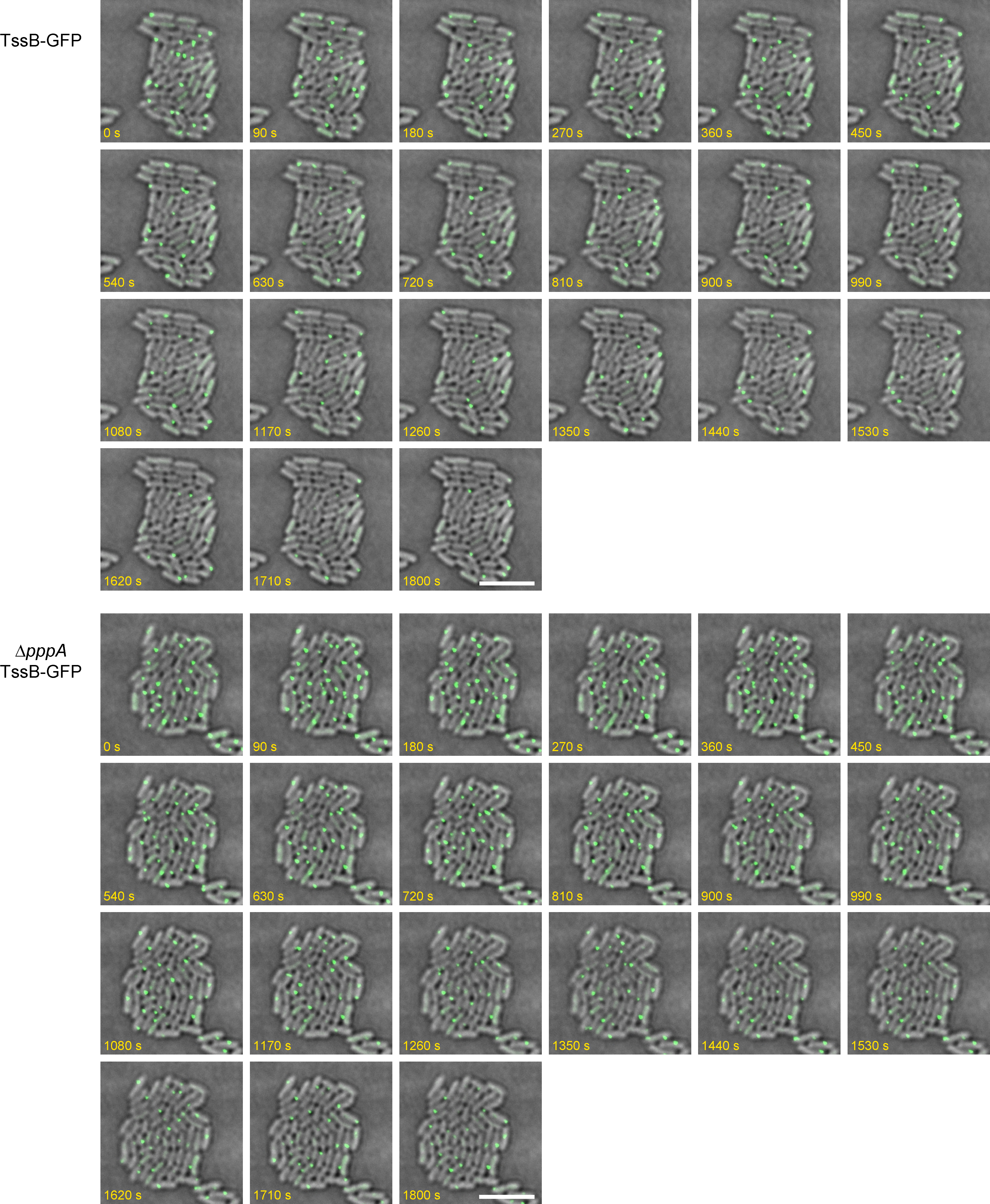

Supplement: S4 Fig — Individual frames showing the whole microcolony for the time-lapse sequences in Fig 2, presented as overlaid images of the TssB-GFP and DIC channels. GFP signal is false-coloured in green and the acquisition time in seconds is indicated. Scale bar, 5 μm. (TIF) [file ppat.1007230.s004.tif]

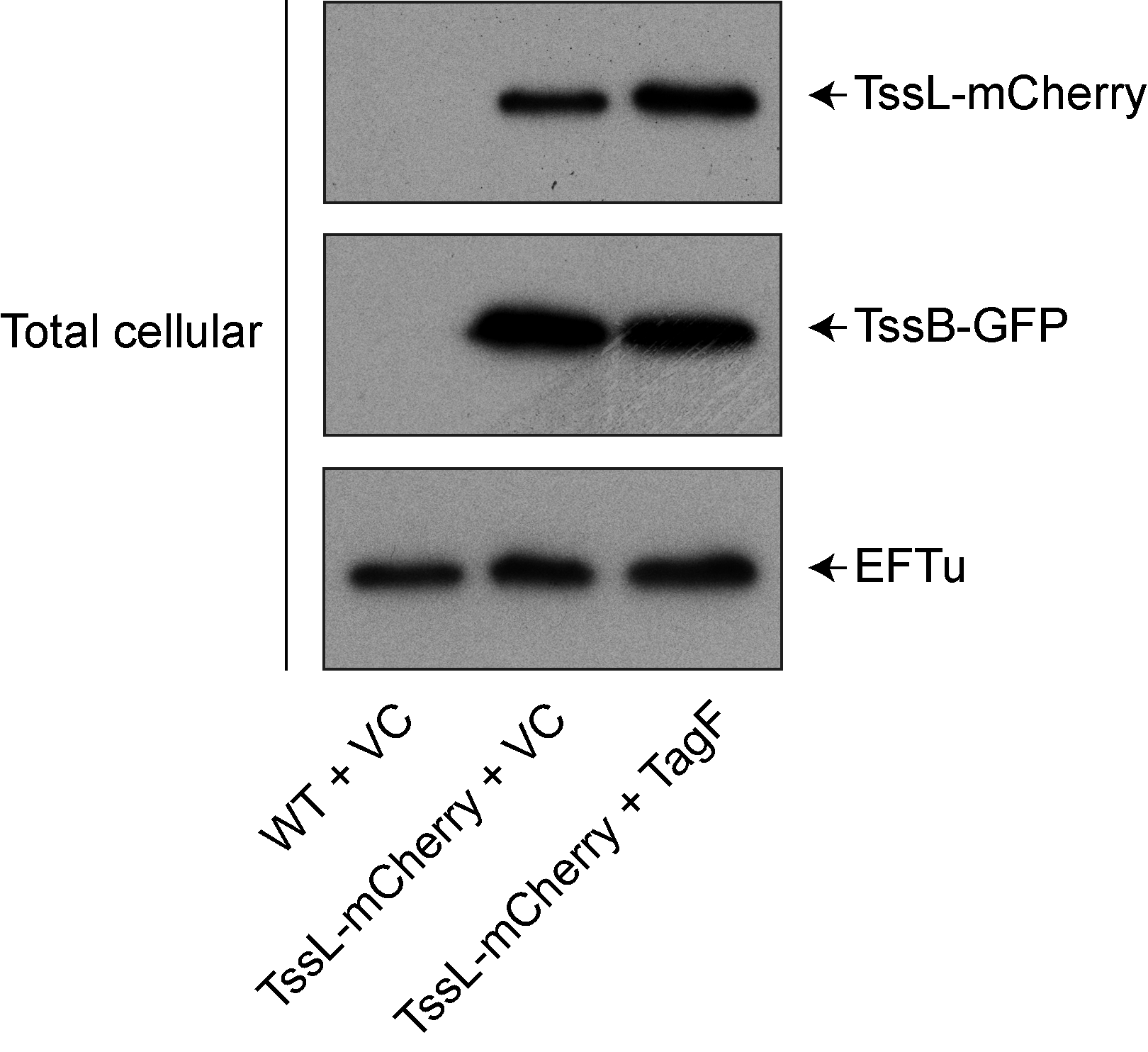

Supplement: S5 Fig — Immunoblot detection of TssL-mCherry, TssB-GFP and control housekeeping protein EFTu in whole cell extracts of wild type S. marcescens Db10 (WT) or the strain expressing TssL-mCherry and TssB-GFP, carrying either the vector control plasmid (+VC, pSUPROM) or a plasmid directing the expression of tagF in trans (+TagF, pSC701). (TIF) [file ppat.1007230.s005.tif]

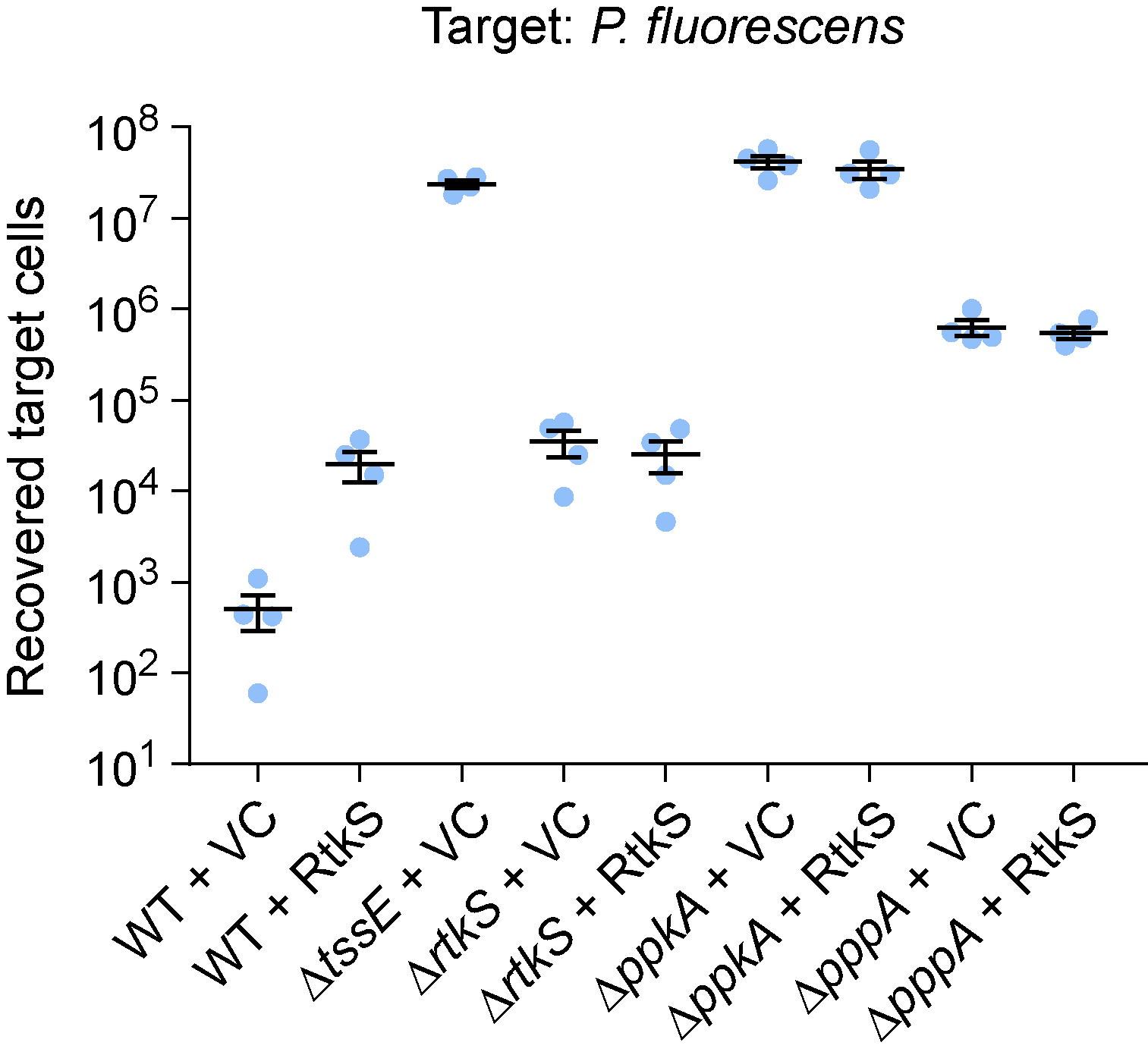

Supplement: S6 Fig — T6SS-dependent anti-bacterial activity as determined by recovery of target organism P. fluorescens following co-culture with wild type (WT) or mutant (ΔtssE, ΔrtkS, ΔppkA and ΔpppA) strains of S. marcescens Db10, carrying either the vector control plasmid (+VC, pSUPROM) or a plasmid directing the expression of rtkS in trans (+ RtkS, pSC590). Individual data points are overlaid with mean +/- SEM (n = 4). (TIF) [file ppat.1007230.s006.tif]

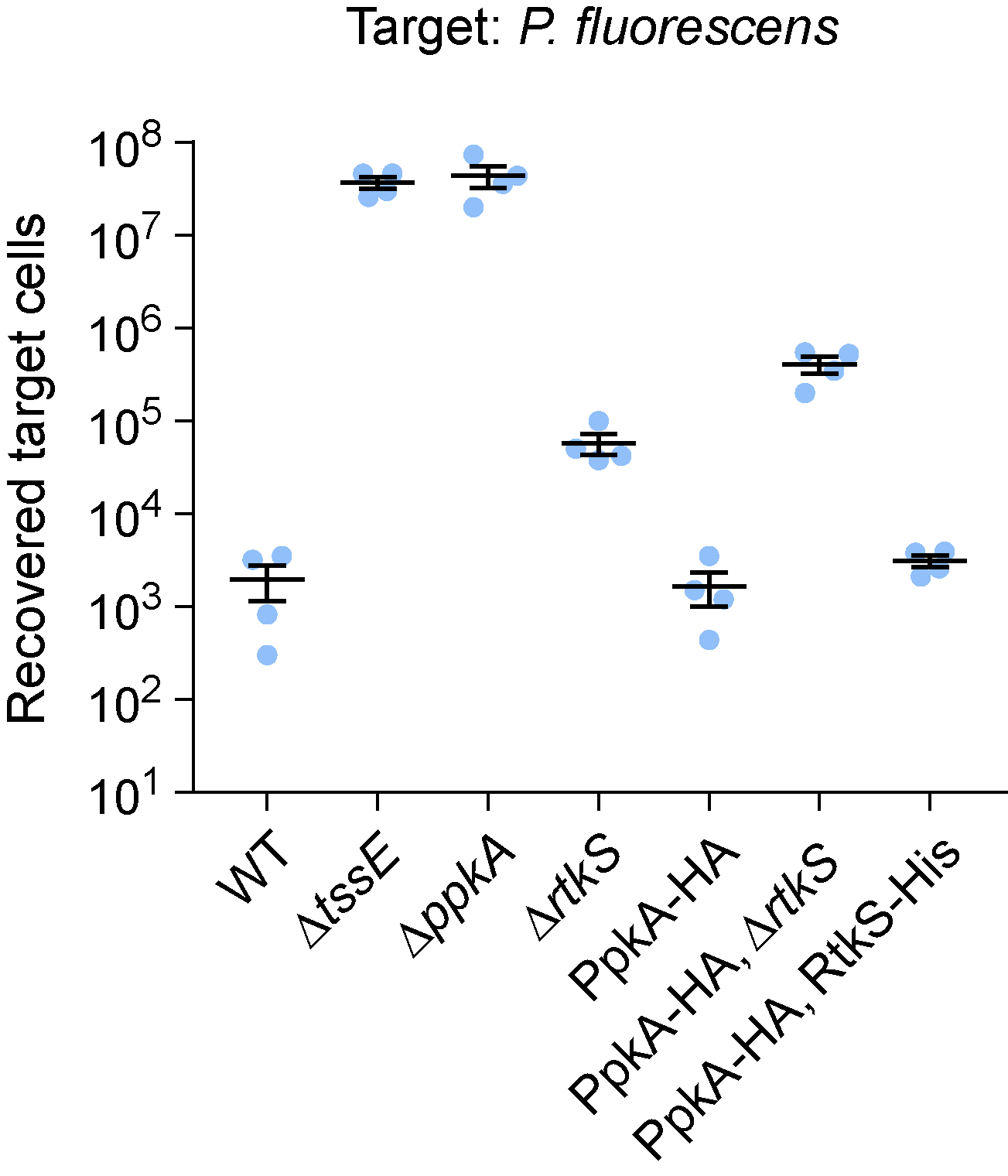

Supplement: S7 Fig — T6SS-dependent anti-bacterial activity as determined by recovery of target organism P. fluorescens following co-culture with wild type (WT) or mutant (ΔtssE, ΔppkA, ΔrtkS, PpkA-HA, PpkA-HA ΔrtkS and PpkA-HA RtkS-His) strains of S. marcescens Db10. Individual data points are overlaid with mean +/- SEM (n = 4). (TIF) [file ppat.1007230.s007.tif]
