# Supplementary material for: Killing with proficiency: Integrated post-translational regulation of an offensive Type VI secretion system
Source: PLoS Pathog. 2018 Jul 27;14(7):e1007230. doi: 10.1371/journal.ppat.1007230 (PMC6082577; doi:10.1371/journal.ppat.1007230)
Supplement: S1 Table — (PDF) [file ppat.1007230.s010.pdf]

S1 Table. Bacterial Strains and Plasmids.

Name	Description	Source / Reference
Strains		
Serratia marcescens		
Db10	Wild type	[1]
SJC11	Db10 Δ*tssE* (Δ*SMDB11_2271*)	[2]
SJC19	Db10 Δ*pppA* (Δ*SMDB11_2268*)	[3]
SJC25	Db10 Δ*ppkA* (Δ*SMDB11_2275*)	[3]
KK2	Db10 Δ*fha* (Δ*SMDB11_2267*)	[3]
MJF16	Db10 *fha-HA* (encodes Fha-HA at normal chromosomal location)	[3]
SJC48	Db10 *fha-HA*, Δ*ppkA*	[3]
SJC49	Db10 *fha-HA*, Δ*pppA*	[3]
KK3	Db10 Δ*tagF* (Δ*SMDB11_2256*)	This study
FRC01	Db10 Δ*ppkA*Δ*tagF*	This study
FRC65	Db10 Δ*pppA*Δ*tagF*	This study
FRC03	Db10 Δ*fha*Δ*tagF*	This study
KB01	Db10 Δ*rtkS* (Δ*SMDB11_2269*), (or Δ*rtkS*)	This study
JJW01	Db10 Δ*ppkA*Δ*rtkS*	This study
JJW02	Db10 Δ*pppA*Δ*rtkS*	This study
JJW03	Db10 Δ*tagF*Δ*rtkS*	This study
FRC63	Db10 *fha-HA*, Δ*rtkS*	This study
GM127	Db10 Δ*ppkA*Δ*tagF*Δ*rtkS*	This study
SAN207	Db10 *tssB-gfpmut2* (encodes TssB-GFP translational fusion at the normal chromosomal location; TssB, SMDB11_2258)	[4]
SAN210	Db10 *tssB-gfpmut2, tssL-mCherry* (encodes TssB-GFP and TssL-mCherry translational fusions at the normal chromosomal locations)	[4]
SAN164	Db10 *fha-mcherry* (encodes Fha-mCherry translational fusion at the normal chromosomal location)	This study
AO14	Db10 *tssB-gfpmut2*, *fha-mcherry*	This study
AO15	Db10 *tssB-gfpmut2*, *fha-mcherry*, Δ*ppkA*	This study
AO16	Db10 *tssB-gfpmut2*, *fha-mcherry*, Δ*tagF*	This study
AO18	Db10 *tssB-gfpmut2*, *fha-mcherry*, Δ*ppkA*Δ*tagF*	This study
AO19	Db10 *tssB-gfpmut2*, Δ*fha*Δ*tagF*	This study
AO21	Db10 *tssB-gfpmut2*, *fha-mcherry*, Δ*pppA*	This study
AO22	Db10 *tssB-gfpmut2*, *fha-mcherry*, Δ*pppA*Δ*tagF*	This study
JJW06	Db10 *tssB-gfpmut2*, *fha-mcherry*, Δ*rtkS*	This study
GM83	Db10 RtkS-His (encodes RtkS [SMDB11_2269] with a C-terminal His_6 tag at the native chromosomal location)	[5]
GM124	Db10 PpkA-HA (encodes PpkA [SMDB11_2275] with a C-terminal HA tag at the native chromosomal location)	This study
GM125	Db10 PpkA-HA, Δ*rtkS*	This study
GM126	Db10 PpkA-HA, RtkS-His	This study
SJC17	Sm-resistant derivative of *S. marcescens* ATCC 274	[2]

Pseudomonas fluorescens		
KT02	Sm-resistant derivative of *P. fluorescens* 55	[2]
Escherichia coli		
MC4100	Model K-12 strain; Sm-resistant (*rpsL150*)	[6]
CC118λ*pir*	Cloning host and donor strain for pKNG101-derived allelic exchange plasmids (λ*pir*)	[7]
HH26 pNJ5000	Mobilizing strain for conjugal transfer	[8]
	MG1655 Δ*cyaA*	F. Sargent
Plasmids		
pKNG101	Suicide vector for allelic exchange (Sm^R *sacBR mobRK2 oriR6K*)	[9]
pSUPROM	Vector for constitutive expression of cloned genes under the control of the *E. coli tat* promoter (Kn^R)	[10]
pUT18	Bacterial Two Hybrid plasmid (for fusion of protein of interest with C-terminal T18 fragment of CyaA; Ap^R)	[11]
pT25	Bacterial Two Hybrid plasmid (for fusion of protein of interest with N-terminal T25 fragment of CyaA; Cm^R)	[12]
pSC571	pKNG101-derived allelic exchange plasmid for the generation of chromosomal in-frame Δ*SMDB11_2269* (Δ*2269*) deletion	This study
pSC904	pKNG101-derived allelic exchange plasmid for the generation of chromosomal in-frame Δ*SMDB11_2267* (Δ*fha*) deletion	This study
pSC906	pKNG101-derived allelic exchange plasmid for the generation of chromosomal in-frame Δ*SMDB11_2256* (Δ*tagF*) deletion	This study
pSC1712	pKNG101-derived allelic exchange plasmid for the generation of chromosomal in-frame Δ*SMDB11_2268* (Δ*pppA*) deletion in strains carrying the *fha-mcherry* fusion	This study
pSAN58	pKNG101-derived allelic exchange plasmid for the generation of chromosomal in-frame *fha-mCherry* fusion	This study
pSC833	pKNG101-derived allelic exchange plasmid for the generation of chromosomal in-frame *ppkA-HA* fusion	This study
pSC590	pSUPROM-derived plasmid for constitutive expression of *SMDB11_2269* (*rtkS*)	This study
pSC701	pSUPROM-derived plasmid for constitutive expression of *tagF* (*SMDB11_2256)*	This study
pSC812	pSUPROM-derived plasmid for constitutive expression of *ppkA* (*SMDB11_2275)*	[3]
pSC591	Coding sequence for SMDB11_2269 (amino acids 20-328) in pUT18	This study
pSC592	Coding sequence for SMDB11_2269 (amino acids 20-328) in pT25	This study
pSC593	Coding sequence for $PpkA^P$ (SMDB11_2275; amino acids 363-482) in pUT18	This study
pSC594	Coding sequence for $PpkA^P$ (SMDB11_2275; amino acids 363-482) in pT25	This study

References:

1. Flyg C, Kenne K, Boman HG. Insect pathogenic properties of *Serratia marcescens*: phage-resistant mutants with a decreased resistance to *Cecropia* immunity and a decreased virulence to *Drosophila*. Journal of General Microbiology. 1980;120(1):173-81. doi: 10.1099/00221287-120-1-173. PubMed PMID: 7012273.

2. Murdoch SL, Trunk K, English G, Fritsch MJ, Pourkarimi E, Coulthurst SJ. The opportunistic pathogen *Serratia marcescens* utilizes type VI secretion to target bacterial competitors. J Bacteriol. 2011;193(21):6057-69. doi: 10.1128/JB.05671-11. PubMed PMID: 21890705; PubMed Central PMCID: PMC3194891.

3. Fritsch MJ, Trunk K, Diniz JA, Guo M, Trost M, Coulthurst SJ. Proteomic Identification of Novel Secreted Antibacterial Toxins of the *Serratia marcescens* Type VI Secretion System. Mol Cell Proteomics. 2013;12(10):2735-49. Epub 2013/07/12. doi: 10.1074/mcp.M113.030502. PubMed PMID: 23842002; PubMed Central PMCID: PMC3790287.

4. Gerc AJ, Diepold A, Trunk K, Porter M, Rickman C, Armitage JP, et al. Visualization of the *Serratia* Type VI Secretion System Reveals Unprovoked Attacks and Dynamic Assembly. Cell Rep. 2015;12(12):2131-42. doi: 10.1016/j.celrep.2015.08.053. PubMed PMID: 26387948; PubMed Central PMCID: PMC4594159.

5. Mariano G, Monlezun L, Coulthurst SJ. Dual Role for DsbA in Attacking and Targeted Bacterial Cells during Type VI Secretion System-Mediated Competition. Cell Rep. 2018;22(3):774-85. doi: 10.1016/j.celrep.2017.12.075. PubMed PMID: 29346773; PubMed Central PMCID: PMC5792426.

6. Casadaban MJ, Cohen SN. Lactose genes fused to exogenous promoters in one step using a Mu-lac bacteriophage: in vivo probe for transcriptional control sequences. Proc Natl Acad Sci USA. 1979;76(9):4530-3. PubMed PMID: 159458; PubMed Central PMCID: PMC411611.

7. Herrero M, de Lorenzo V, Timmis KN. Transposon vectors containing non-antibiotic resistance selection markers for cloning and stable chromosomal insertion of foreign genes in gram-negative bacteria. J. Bacteriol. 1990;172(11):6557-67. PubMed PMID: 2172216; PubMed Central PMCID: PMC526845.

8. Grinter NJ. A broad-host-range cloning vector transposable to various replicons. Gene. 1983;21(1-2):133-43. Epub 1983/01/01. PubMed PMID: 6301943.

9. Kaniga K, Delor I, Cornelis GR. A wide-host-range suicide vector for improving reverse genetics in gram-negative bacteria: inactivation of the *blaA* gene of *Yersinia enterocolitica*. Gene. 1991;109(1):137-41. Epub 1991/12/20. PubMed PMID: 1756974.

10. Jack RL, Buchanan G, Dubini A, Hatzixanthis K, Palmer T, Sargent F. Coordinating assembly and export of complex bacterial proteins. EMBO J. 2004;23(20):3962-72. PubMed PMID: 15385959

11. Karimova G, Ullmann A, Ladant D. Protein-protein interaction between *Bacillus stearothermophilus* tyrosyl-tRNA synthetase subdomains revealed by a bacterial two-hybrid system. J Mol Microbiol Biotechnol. 2001;3(1):73-82. Epub 2001/02/24. PubMed PMID: 11200232.

12. Karimova G, Pidoux J, Ullmann A, Ladant D. A bacterial two-hybrid system based on a reconstituted signal transduction pathway. Proc Natl Acad Sci USA. 1998;95(10):5752-6. Epub 1998/05/20. PubMed PMID: 9576956; PubMed Central PMCID: PMC20451.
