# Supplementary material for: Killing with proficiency: Integrated post-translational regulation of an offensive Type VI secretion system
Source: PLoS Pathog. 2018 Jul 27;14(7):e1007230. doi: 10.1371/journal.ppat.1007230 (PMC6082577; doi:10.1371/journal.ppat.1007230)
Supplement: S2 Table — (PDF) [file ppat.1007230.s011.pdf]

S2 Table. Oligonucleotide primers for plasmid construction.

Plasmid	Sequence of relevant primers (5'-3')[1]	Description
pSC571	TATATCTAGAGCATTCGTCAGCACATCACC	Forward primer to clone upstream region of SMDB11_2269 in pKNG101 (*Xba*I)
	TATAAAGCTTCCAATATTTCATAGTGAACCTTGCTG	Reverse primer to clone upstream region of SMDB11_2269 in pKNG101 (*Hin*dIII)
	TATAAAGCTTGCCACTCACTGATTATAAGGAAAGC	Forward primer to clone downstream region of SMDB11_2269 in pKNG101 (*Hin*dIII)
	TATAGGGCCCGCAGTTGGCATACCTGTTCG	Reverse primer to clone downstream region of SMDB11_2269 in pKNG101 (*Apa*I)
pSC904	TGTATCTAGAAGGTGAAAAACTGGCTGAACAG	Forward primer to clone upstream region of SMDB11_2267 in pKNG101 (*Xba*I)
	TAATGGATCCAAATCGCATAAGGCATTCCTGC	Reverse primer to clone upstream region of SMDB11_2267 in pKNG101 (*Bam*HI)
	TATAGGATCCGACTCACAGACCAAACCGGAC	Forward primer to clone downstream region of SMDB11_2267 in pKNG101 (*Bam*HI)
	TATAGGGCCCATATGCAGCGACTGTTCC	Reverse primer to clone downstream region of SMDB11_2244 in pKNG101 (*Apa*I)
pSC906	TATATCTAGAAAGTGACGCCGGACGATCTG	Forward primer to clone upstream region of SMDB11_2256 in pKNG101 (*Xba*I)
	TAATGGATCCAGTGCTCATGGTCATGTCCTTTC	Reverse primer to clone upstream region of SMDB11_2256 in pKNG101 (*Bam*HI)
	TGGTGGATCCCAAATGTTTGATTAACATCGTTCGTTG	Forward primer to clone downstream region of SMDB11_2256 in pKNG101 (*Bam*HI)
	TATAGGGCCCAATGGCGCTCAGGGTGTTG	Reverse primer to clone downstream region of SMDB11_2256 in pKNG101 (*Apa*I)
pSC1712	TATATCTAGACCCCGACTACTTGAAGCTGTCC	Forward primer to clone upstream region of SMDB11_2268 in fha-mCherry background in pKNG101 (*Xba*I)
	TATAAAGCTTGGTGATATTCATTACGCGTCCG	Reverse primer to clone upstream region of SMDB11_2268 in fha-mCherry background in pKNG101 (*Hin*dIII)
	TGTGAAGCTTTTTCTGAGCCGCGACTGAG	Forward primer to clone dowstream region of SMDB11_2268 in pKNG101 (*Hin*dIII)
	TATAGGGCCCTGTTGCAGCGTGAC	Reverse primer to clone downstream region of SMDB11_2268 in pKNG101 (*Apa*I)
pSAN58	TATATCTAGAAAGTGGCGACGCATTGG	Forward primer to amplify SMDB11_2267 3' end for generation of Fha-mCherry fusion construct by overlap PCR (*Xba*I)
	CCATGGTGGCGACCGGTGCTCCTGCTCCCGCGTCCGGTTTGGTC	Reverse primer to amplify SMDB11_2267 3' end for generation of Fha-mCherry fusion construct by overlap PCR
	GACCAAACCGGACGCGGGAGCAGGAGCACCGGTCGCCACCATGG	Forward primer to amplify *mCherry* gene from pmCherry-N1 (Clontech) for generation of Fha-mCherry fusion construct by overlap PCR
	CGGTTTGGTCTGTGAGTCTTTCTTGTACAGCTCGTCCATGCC	Reverse primer to amplify *mCherry* gene for generation of Fha-mCherry fusion construct by overlap PCR
	GGCATGGACGAGCTGTACAAGAAAGACTCACAGACCAAACCG	Forward primer to amplify SMDB11_2267 downstream region for generation of Fha-mCherry fusion construct by overlap PCR

	TATAGGGCCCGCAGCGACTGTTCCAGTTC	Reverse primer to amplify SMDB11_2267 downstream region for generation of Fha-mCherry fusion construct by overlap PCR (*Apa*I)
pSC833	TATATCTAGACGCAGACCATCGATGAAATG	Forward primer to clone 3’ end of SMDB11_2275 in pKNG101 (*Xba*I)
	ATATAAGCTTTA**TGCATAATCAGGAACATCATAAGGATA**AC	Reverse primer to clone 3’ end of SMDB11_2275 incorporating C-terminal **HA tag**[2] (amplified from pSC814) in pKNG101 (*Hin*dIII)
	ATATAAGCTTCCCGTTCTGAAACTCCGC	Forward primer to clone region downstream of SMDB11_2275 in pKNG101 (*Hin*dIII)
	TATAGGGCCCAAACTGCTTTCGTTGTACTGCACG	Reverse primer to clone region downstream of SMDB11_2275 in pKNG101 (*Apa*I)
pSC590	TATAAGATCTATGAAATATTGGATGCCGGG	Forward primer to clone SMDB11_2269 in pSUPROM (*Bgl*II)
	TATATCTAGAGCTTTCCTTATAATCAGTGAGTGGC	Reverse primer to clone SMDB11_2269 in pSUPROM (*Xba*I)
pSC701	TATAGGATCCATGAGCACTGATTCAAGCGC	Forward primer to clone SMDB11_2256 in pSUPROM (*Bam*HI)
	TATATCTAGATTAATCAAACATTTGCGGGTACTG	Reverse primer to clone SMDB11_2256 in pSUPROM (*Xba*I)
pSC591	TATAGCATGCGTAAAGAGGAGGTGCCATGAACTATCGCCTGGTTTACTCC	Forward primer to clone SMDB11_2269 (amino acids 20-328) in pUT18 (*SphI*)
	TATATCTAGAGAGTGAGTGGCGCCGTTAACC	Reverse primer to clone SMDB11_2269 (amino acids 20-328) in pUT18 (*Xba*I)
pSC593	TATAGCATGCGTAAAGAGGAGGTGCCATGGGCGGCGAGGACGATACC	Forward primer to clone SMDB11_2275 (amino acids 363-482) in pUT18 (*Sph*I)
	TATATCTAGAGAACCGGCGGTGGCCGGATTGAC	Reverse primer to clone SMDB11_2275 (amino acids 363-482) in pUT18 (*Xba*I)
pSC592	TATAAGATCTAATGAACTATCGCCTGGTTTACTCC	Forward primer to clone SMDB11_2269 (amino acids 20-328) in pT25 (*Bgl*II)
	TATAGGTACCTCAGTGAGTGGCGCCGTTAACC	Reverse primer to clone SMDB11_2269 (amino acids 20-328) in pT25 (*Kpn*I)
pSC594	TATAGGATCCAGGCGGCGAGGACGATACC	Forward primer to clone SMDB11_2275 (amino acids 363-482) in pUT25 (*Bam*HI)
	TATAGGTACCTTAACCGGCGGTGGCCGGATTGAC	Reverse primer to clone SMDB11_2275 (amino acids 363-482) in pUT25 (*Kpn*I)

[1]Incorporated restriction sites for cloning into the respective vector are underlined.
[2]HA (haemagglutinin) tag shown in bold, immediately follows final amino acid of SMDB11_2275 (PpkA).
